# CENP-C/H/I/K/M/T/W/N/L and hMis12 but not CENP-S/X participate in complex formation in the nucleoplasm of living human interphase cells outside centromeres

**DOI:** 10.1371/journal.pone.0192572

**Published:** 2018-03-06

**Authors:** Christian Hoischen, Sibel Yavas, Thorsten Wohland, Stephan Diekmann

**Affiliations:** 1 Molecular Biology, Leibniz Institute on Aging–Friz-Lipmann-Institute (FLI), Jena, Germany; 2 Departments of Biological Sciences and Chemistry and Centre of Bioimaging Sciences, Lee Wee Kheng Buildung, National University of Singapore, Singapore, Singapore; Florida State University, UNITED STATES

## Abstract

Kinetochore proteins assemble onto centromeric chromatin and regulate DNA segregation during cell division. The inner kinetochore proteins bind centromeres while most outer kinetochore proteins assemble at centromeres during mitosis, connecting the complex to microtubules. Here, we measured the co-migration between protein pairs of the constitutive centromere associated network (CCAN) and hMis12 complexes by fluorescence cross-correlation spectroscopy (FCCS) in the nucleoplasm outside centromeres in living human interphase cells. FCCS is a method that can tell if in living cells two differently fluorescently labelled molecules migrate independently, or co-migrate and thus are part of one and the same soluble complex. We also determined the apparent dissociation constants (K_d_) of the hetero-dimers CENP-T/W and CENP-S/X. We measured co-migration between CENP-K and CENP-T as well as between CENP-M and CENP-T but not between CENP-T/W and CENP-S/X. Furthermore, CENP-C co-migrated with CENP-H, and CENP-K with CENP-N as well as with CENP-L. Thus, in the nucleoplasm outside centromeres, a large fraction of the CENP-H/I/K/M proteins interact with CENP-C, CENP-N/L and CENP-T/W but not with CENP-S/X. Our FCCS analysis of the Mis12 complex showed that hMis12, Nsl1, Dsn1 and Nnf1 also form a complex outside centromeres of which at least hMis12 associated with the CENP-C/H/I/K/M/T/W/N/L complex.

## Introduction

Chromosome segregation is executed by a conserved molecular machinery which contains a large number of subunits and recruits many additional regulatory proteins (recently reviewed in [1–5]). A multi-protein complex, the “kinetochore”, assembles onto centromeric chromatin [6–15]. During mitosis, the kinetochore mediates the interaction between DNA and the mitotic spindle [16–28] (reviewed in [29]). Kinetochores are built from an inner layer, directly contacting centromeric chromatin, and an outer layer, binding to the spindle microtubules. The inner kinetochore controls outer kinetochore assembly [30–32], influences microtubule binding [31,33], and contributes to epigenetic specification of centromeres [21–26,34–37].

Although centromeres are directly embedded in chromatin, specific DNA sequences are neither necessary nor sufficient for centromere function. Instead, the centromere seems to be epigenetically defined, and its location inherited, by the H3 histone variant CENP-A, which replaces canonical H3 in centromeric nucleosomes [38–56]. CENP-A propagates centromere identity and nucleates kinetochore formation [57–63].

The 16-subunit Constitutive Centromere-Associated Network (CCAN) localizes to centromeres throughout the cell cycle and provides the foundation for outer kinetochore assembly on CENP-A-containing chromatin [18,35,64–72]. Also non-coding satellite RNA is involved in centromere regulation and CENP-A loading to the centromere [73,74]. Two CCAN proteins, CENP-C and CENP-N, bind CENP-A nucleosomes directly [48,54,70,75–82] while CENP-C and CENP-T associate with proteins of the kinetochore microtubule binding interface [21–26,78,83–85]. Thus, the CCAN proteins establish a link between the centromere and the outer kinetochore.

The CCAN proteins can be grouped into five sub-units: CENP-C, CENP-L/N, CENP-H/I/K/M, CENP-T/W/S/X, and CENP-O/P/Q/U/R [35,38,68,69,75,86–92]. The relationships between these proteins were extensively studied [12–15,30,31,35,37,50,68,70,75,76,79,83,88–90,92–96]. CENP-H, CENP-I and CENP-K form a complex [31, 32,35] with CENP-M [92]. The complex formed by CENP-A and CENP-C/H/I/K/M/L/N seems to be basic for kinetochore function [15]. The binding of CENP-T/W/S/X to CENP-A requires CENP-C and the presence of the CENP-H/I/K/M complex [15,92]. The CENP-T/W/S/X complex contains proteins with histone-fold domains that bind DNA and might form a nucleosome-like structure [52,70,91,97,98]. Several interactions among CCAN proteins have been identified in *S*. *cerevisiae* and *S*. *pombe* [11,23,64,65,67,94,99–106] as well as in other organisms [107], indicating a strong evolutionarily conserved homology of kinetochore assembly in many [108] however no or very little homology in a few other organisms [109,110].

CENP-I is required to generate a stable association of the RZZ complex (formed by Rod, ZW10 and Zwilch) and Mad1 with kinetochores and also inhibits their removal by dynein [111,112]. The CENP-H/I/K/M proteins are proximal to the CENP-N/L complex but also to the subunits of the CENP-T/W/X/S and CENP-O/P/Q/U complexes [35,66,68,69,113]. The CENP-O/P/Q/U complex interacts with CENP-R [88,90,114,115] and is involved in microtubule binding and spindle checkpoint control [10]. CENP-T interacts directly with the Ndc80 complex contributing to outer kinetochore assembly [18,21–24,78,83–85] while CENP-C binds the Mis12 complex [25,26]. The CENP-S/X hetero-dimer is not essential for mitosis but plays a role in kinetochore stabilisation [89,97].

At the centromere, the CCAN sub-units show multiple interactions with other sub-complexes, centromeric nucleosomes [15], and/or DNA [12] and RNA [73]. Kinetochore assembly depends on the combination of these interactions. The contributions of these interactions vary during the cell cycle [14,79,116]. Thus, the extensive network of interactions formed between sub-units plays a critical role in providing a specific and robust foundation for the chromosome segregation machinery [12,15].

We speculated that these protein-protein interactions may be established partly already in the nucleoplasm at non-centromeric sites, before these proteins assemble at the centromere. Indeed, for some CCAN proteins we recently found that they form diffusible complexes in the nucleoplasm before binding to the centromere: by fluorescence cross-correlation spectroscopy (FCCS) we detected a strong association for CENP-S and CENP-X [97], a slightly weaker interaction for CENP-O and CENP-P [90], and a weak association for CENP-R and CENP-Q [90]. FCCS analysis can identify if two differently labeled molecules move together (in a complex) or independently, indicating direct or indirect protein-protein interaction. Fluorescence correlation spectroscopy (FCS) analyses diffusing particles (summarized in [117]) by monitoring the fluorescence fluctuations created when molecules diffuse through a diffraction limited confocal detection volume. Photons emitted from the fluorescent particles are counted continuously over time. While the fluctuation amplitude depends on particle concentration and brightness, its frequency contains information on the diffusion times of the fluorescent particles. For quantitative evaluation, the fluctuation frequency is correlated with a time-shifted replica of itself (autocorrelation). The amplitude of the autocorrelation curve is inversely proportional to the average number of fluorescent molecules in the confocal volume. By monitoring the diffusion coefficient FCS can determine molecular association and binding affinities. However, the diffusion coefficient is inversely proportional to the hydrodynamic radius of a particle and thus weakly dependent on its overall mass. Therefore, it is difficult to determine the association of similar sized particles. This limitation can be circumvented by fluorescence cross-correlation spectroscopy (FCCS). When two differently labeled molecules are measured in the same volume and correlated by cross-correlation, the analysis can tell if both molecules move together (in a complex) or independently, enabling the deduction of dissociation constants (K_d_) of binding reactions *in vivo* [117].

Here, we extend our FCCS studies in living human cells [90, 97] to a number of other CCAN and hMis12 complex proteins. We found that in the nucleoplasm outside kinetochores CENP-T co-migrates with CENP-K and CENP-M. The *in vivo* interactions between CENP-T and CENP-W as well as between CENP-S and CENP-X are studied in detail. We found that a large fraction of the CCAN proteins CENP-C/H/I/K/M/T/W/N/L but not CENP-S/X interact with one another *in vivo* outside centromeres, supporting the findings of Weir et al. [15].

## Materials and methods

### Plasmids

Cloning of CENP-M, -N, -S, -T and CENP-X have been described elsewhere [71,97,118]. Full length coding sequences of CENP-L (IRAUp969E0882D, RZPD Berlin), CENP-W (IRATp970A07105D, imaGenes Berlin), hMis12 (IRAUp969C0611D6, RZPD Berlin), SPC24 (IMAGp958K072621Q, RZPD Berlin), SPC25 (IRAUp969F0887D, RZPD Berlin), Nsl1 (pIC79, Iain Cheeseman), Dsn1 (pIC80, Iain Cheeseman), Nnf1 (pIC81, Iain Cheeseman), CENP-C/H/I [116] were amplified by PCR (Expand high fidelity^PLUS^ PCR System, Roche, Penzberg, Germany) using primers incorporating flanking attB recombination sites and transferred into vector pDONR221 by BP recombination reaction (Invitrogen, Carlsbad, CA, USA). Genes were then transferred by LR recombination reactions into modified pFP-C and pFP-N (BD Biosciences, Clontech, Palo Alto, CA, USA) based Destination vectors. The fusion constructs have either short (FP-(s)-CENP) or long (FP-(l)-CENP) linkers. In FP-(s)-CENP constructs, the amino acid (aa) linker between the two fused proteins is SGTSLYKKAGFENLYFQGAT, whereas in FP-(l)-CENP constructs the linker is extended to SGTSLYKKAGFGGSAGGSGSGSSGGSSGASGTGTAGGTGSGSGTGSGG.

### Cell lines and transfection

HEp-2 and U2OS cells were cultured in DMEM with 10% FCS as previously described [79,119]. For the dual color fluorescence cross-correlation spectroscopy (DC-FCCS) experiments, appropriate constructs were transfected into HEp-2 and U2OS cells (ATCC, Manassas, VA, USA) by electroporation and interphase cells were assayed by DC-FCCS after 24–48 hrs.

For single-wavelength fluorescence cross-correlation spectroscopy (SW-FCCS), HEK293 cells (ATCC) were cultivated in Dulbecco`s modified Eagles’s medium DMEM (Hyclone, GE Healthcare, UK) supplemented with 10% FBS (Hyclone fetal bovine serum, GE Healthcare, UK) and 1% PS, penicillin G and streptomycin (PAA, Austria) at 37°C in 5% CO_2_ atmosphere. Appropriate plasmids were transfected into the cells by electroporation (Neon^R^ Transfection system, Life Technologies, Carlsbad, CA). Around 90% confluent cells were washed twice with 5 ml 1×PBS (phosphate buffer saline), treated with 0.5 ml Trypsin for 1 min and re-suspended in 5 ml DMEM culture medium. Cells (1×10^6^) were centrifuged for 3 min, re-suspended in R buffer and transfected according to the manufacturer’s protocol. The transfected cells were seeded in DMEM and 10% FBS culture medium on glass coverslips (MatTEK, Ashland, US) and kept at 37°C in 5% CO_2_ atmosphere overnight. Before starting the experiments, the cells were washed with 1×PBS and maintained in 1 ml 1×PBS.

### Fluorescence cross-correlation spectroscopy (FCCS)

#### Dual color fluorescence cross-correlation spectroscopy (DC-FCCS)

Dual Color FCCS analyses [120,121] were performed at 37°C on an LSM 710 Confocor3 microscope (Carl Zeiss, Jena, Germany) using a C Apochromat 40x/1.2 NA water objective. U2OS cells were double transfected with vectors for the simultaneous expression of EGFP and mCherry fusion proteins and analysed. In interphase cells expressing both fusion proteins at relatively low and comparable levels, we selected spots for the FCCS measurements in areas of the nucleoplasm which were free of kinetochores. For illumination of the EGFP-fusion proteins, we used the 488 nm laser line of a 25 mW Argon/2-laser (Carl Zeiss) and for simultaneous illumination of the mCherry fusion proteins a DPSS 561-10-laser (Carl Zeiss), both at moderate intensities between 0.2 and 0.5%. The detection pinhole was set to a relatively small diameter of 40 μm (corresponding to about 0.8 airy units). After passing a dichroic beam splitter (NTF 565, standard filter of LSM710), the emission of mCherry was recorded in channel 1 through a BP-IR 615–680 nm bandpath filter by an APD (avalanche photodiode), whereas the emission of EGFP was simultaneously recorded in channel 2 through a BP-IR 505–540 nm bandpath filter by a second APD. Before each measurement, we analysed possible crosstalk between the channels and used only cells without or with very little crosstalk. Furthermore, before data sampling, the selected focal volume was bleached destroying the fluorescence of immobile fluorophores. Thus, after bleaching, only mobile fluorophores (like tagged CENPs) can contribute to the DC-FCCS signal. In addition, measurements with autocorrelation values below 1.05 for both, the mRFP channel as well as the EGFP channel, were not further analysed. For the measurements, 10 time series of 10 sec each were simultaneously recorded for mCherry and for EGFP. After averaging, the data were superimposed for fitting with the Fit-3Dfree-1C-1Tnw model of the ZEN-software (Carl Zeiss), a diffusion model in three dimensions with a triplet. Applying this procedure, we obtained autocorrelations of channels 1 and 2 as well as the cross-correlation of channels 1 versus channel 2. Before starting a set of experiments, the pinhole position was adjusted to the beam path. As negative control, U2OS cells were transfected with vector pIRES2, separately expressing EGFP and mRFP as single molecules with fluorescence intensities comparable to those in the FCCS analysis with CENP fusion proteins. As a positive control, U2OS cells were transfected with pH-mR-G-C expressing a mRFP-EGFP fusion protein, again with fluorescence intensities comparable to those in the FCCS analysis with CENP fusion proteins.

#### Single-Wavelength Fluorescence Cross-Correlation Spectroscopy (SW-FCCS)

Single-Wavelength Fluorescence Cross-Correlation Spectroscopy (SW-FCCS) setup and calibration was described previously [122,123]. In short, the experiments were performed on a modified Olympus FV300 confocal microscope with a 1.2 NA 60x water immersion objective (UplanApo, Olympus, Japan). EGFP- and mCherry-labelled proteins were excited by a 20 μW Argon ion 514 nm laser line (Melles Griot, Albuquerque, NM, USA). The emission passed a 150 μm pinhole, split by a 560 DCLP emission dichroic mirror (Omega Optical, Brattleboro, VT, USA) followed by the band-pass filters (545AF35 and 615DF45, respectively, Omega Optical, Brattleboro, VT, USA) to the two avalanche photodiodes (SPCM-AQR-14; PerkinElmer, Canada). Auto- and cross-correlation curves were generated by a hardware correlator (Flex02-01D, www.correlator.com, Bridgewater, NJ, USA). The experiments were conducted at 37°C (TempContro 37–2, Pecon, Erbach, Germany) equipped with an objective heating ring (TC-124A, Warner Instruments, Hamden, CT, USA). Each single-point measurement recorded 3 times 25 sec runs in the nucleoplasm. The obtained auto- and cross-correlation functions (ACF and CCF) were fitted with a self-written software in Igor Pro 6.22A (WaveMetrics, Lake Oswego, OR, USA) using a 3-dimensional 1-particle 1-triplet model [124]. The concentration calculations and dimer fraction quantification was conducted by a self-written program in Mathematica 10 (Wolfram Research, Champaign, IL, USA). For the calculation of apparent dissociation constants (*K*_*d*_) the data were corrected for the probability of mCherry to be fluorescent (*p*_*r*_) and for the differences in the effective observation volumes at different wavelength. The detailed protocols for the correction have been published previously [124]. EGFP was assumed to be fully fluorescent (*p*_*g*_ = 1). The green observation volume *V*_*G*_ was 0.54 fl as determined by calibration of the system with Atto488. The red observation volume *V*_*R*_ was determined by the ratio of diffusion times of mCherry/EGFP (τ_*D*,*R*_*/τ*_*D*,*G*_) from the measurement of a tandem EGFP-mCherry construct. The ratio of τ_*D*,*R*_*/τ*_*D*,*G*_ was 1.1, which translated into a *V*_*R*_*/V*_*G*_ ratio of 1.15 (τ_*D*_ is proportional to ω_o_^2^, *V* is proportional to ω_o_^3^), resulting in *V*_*R*_ = 0.62 fl. The overlap of these two observation volumes gave an effective cross-correlation volume *V*_*x*_ of 0.58 femto-litre (fl). These parameters resulted in a *p*_*r*_ = 0.6, implying that slightly more than half of all mCherry fluoresce. The fraction of non-fluorescent mCherry proteins limits the maximum cross-correlation that can be achieved.

## Results and discussion

16 CCAN proteins, forming the sub-complexes CENP-T/W/S/X, CENP-H/I/K/M, CENP-L/N and CENP-P/O/R/Q/U, assemble at the centromere during all phases of the cell cycle. We studied if these proteins and the four proteins of the hMis12 complex form pre-complexes in the nucleoplasm outside centromeres.

Recently we analysed protein interactions between proteins of the CENP-P/O/R/Q/U complex and their interactions with other CCAN proteins [90]. By Yeast-2-Hybrid (Y2H) analysis of these human proteins in *S*. *cerevisiae* we could only detect a weak interaction of CENP-R with itself. However, using a mammalian three-hybrid (F3H) [125,126] assay for protein-protein interactions in living human cells at non-centromeric chromatin locations, we detected the interactions between CENP-O and CENP-P as well as CENP-R, between CENP-U and CENP-P, CENP-R and CENP-Q and between CENP-Q and CENP-R. When analysing the diffusible proteins of the CENP-P/O/R/Q/U complex individually in the nucleoplasm of human cells by dual wavelengths fluorescence cross-correlation spectroscopy (DC-FCCS), we observed co-migration between CENP-O and CENP-P (40–50%) and between CENP-Q and CENP-R (16–20%) (see Table 1) but not between other complex members [90], suggesting that the CENP-P/O/R/Q/U proteins only partially pre-form sub-complexes but not a fivefold complex in the nucleoplasm outside centromeres.

**Table 1:** Cross-correlation by DC-FCCS.

cross-correlation in nucleoplasm				
	mCherry fusion	Measured	Corrected	Measurements/
		(mean±SD %)	(%)	number of cells
CENP-P/O/R/Q/U complex				
EGFP-(s)-CENP-O*	mCherry-(s)-CENP-P*	25±3	40–50	12/12
EGFP-(s)-CENP-R*	mCherry-(s)-CENP-Q*	10±2	16–20	2/2
CENP-H/I/K/M complex				
EGFP-(l)-CENP-M	mCherry-(s)-CENP-K	37±9	56–83	19/12
EGFP-(l)-CENP-M	mCherry-(s)-CENP-I	24±9	30–66	32/18
EGFP-(s)-CENP-I	mCherry-(s)-CENP-M	0	0	12/4
CENP-I-(s)-EGFP	CENP-M-(s)-mCherry	0	0	10/6
EGFP-(s)-CENP-H	CENP-I-(s)-mCherry	22±6	32–58	26/14
EGFP-(s)-CENP-H	mCherry-(s)-CENP-K	31±8	46–78	23/10
EGFP-(s)-CENP-K	mCherry-(s)-CENP-I	20±7	26–54	19/10
CENP-C				
EGFP-(s)-CENP-H	mCherry-(s)-CENP-C	26±7	38–66	20/10
EGFP-(s)-CENP-H	CENP-C-(s)-mCherry	18±10	16–56	11/5
CENP-N/L complex				
EGFP-(l)-CENP-L	mCherry-(s)-CENP-N	23±9	28–64	16/9
EGFP-(l)-CENP-L	mCherry-(s)-CENP-K	18±7	22–50	22/10
EGFP-(s)-CENP-N	mCherry-(s)-CENP-K	0	0	4/2
CENP-K-(s)-EGFP	mCherry-(s)-CENP-N	26±13	26–78	17/8
CENP-T/W/S/X complex				
EGFP-(s)-CENP-X^#^	mCherry-(s)-CENP-S^#^	40±10	60–100	19/12
EGFP-(s)-CENP-X	mCherry-(l)-CENP-S	31±7	48–76	9/7
CENP-T-(s)-EGFP	mCherry-(s)-CENP-W	28±8	40–72	18/12
EGFP-(s)-CENP-T	mCherry-(s)-CENP-S	0	0	14/8
EGFP-(s)-CENP-T	CENP-S-(s)-mCherry	0	0	7/5
EGFP-(s)-CENP-T	mCherry-(l)-CENP-S	0	0	6/4
EGFP-(l)-CENP-T	CENP-S-(l)-mCherry	0	0	6/3
EGFP-(s)-CENP-T	mCherry-(s)-CENP-X	0	0	10/5
EGFP-(l)-CENP-W	CENP-X-(l)-mCherry	0	0	4/2
EGFP-(s)-CENP-M	CENP-T-(s)-mCherry	13±3	20–32	4/2
EGFP-(s)-CENP-M	CENP-T^∆C^-(s)-mCherry	0	0	14/5
CENP-K-(s)-EGFP	CENP-T-(s)-mCherry	31±13	36–88	20/10
hMis12 complex				
EGFP-(s)-Dsn1	mCherry-(s)-Nnf1	22±3	38–50	18/10
EGFP-(s)-Nsl1	mCherry-(s)-Nnf1	18±6	24–48	15/11
EGFP-(s)-Dsn1	mCherry-(s)-Nsl1	24±5	38–58	13/8
hMis12-(s)-EGFP	Nnf1-(s)-mCherry	31±8	46–78	22/10
Nsl1-(s)-EGFP	hMis12-(s)-mCherry	36±11	50–94	19/9
EGFP -(s)-Dsn1	hMis12-(s)-mCherry	32±7	50–78	18/10
hMis12-(s)-EGFP	mCherry-(s)-CENP-K	18±4	28–44	22/10
hMis12-(s)-EGFP	mCherry-(s)-CENP-T	18±4	28–44	17/8
hMis12-(s)-EGFP	CENP-T-(s)-mCherry	19±4	30–46	8/5

Measured and the corrected values (in %) for the CENP or hMis12 complex protein pairs tagged by EGFP or mCherry at the N- or C-terminal end. (s): short linker, (l): long linker (see Materials & Methods). The original data behind “means” and “SD” are listed in S1 Table. Since cross-correlation values under-estimate the factual percentage of co-migrating molecules approximately by a factor two, due to various effects including non-fluorescent proteins and differences in the size of the diffraction limited volume for the two measured wavelengths, the values measured with DC-FCCS had to be corrected (see main text and reference [124]).

*) [83]

^#^) [90]

Here we analysed potential protein-protein interactions among further CCAN proteins in the nucleoplasm of living human interphase cells outside centromeres. We double-transfected human HEp-2 cells so that they express two CCAN proteins, terminally tagged with EGFP in the one and mCherry in the other case. By DC-FCCS we then measured the mobility of both tagged proteins in a very small confocal volume (ca. 1 fl, about half the size of *E*. *coli*) and, by cross-correlation of the fluorescence signal, we determined if in the focal volume the two proteins co-migrate which would indicate both proteins being part of a di- or multimeric complex [90,117,127–129]. FCCS possesses single-molecule sensitivity and can be conducted at concentrations in the nanomolar range.

**Fig 1:**
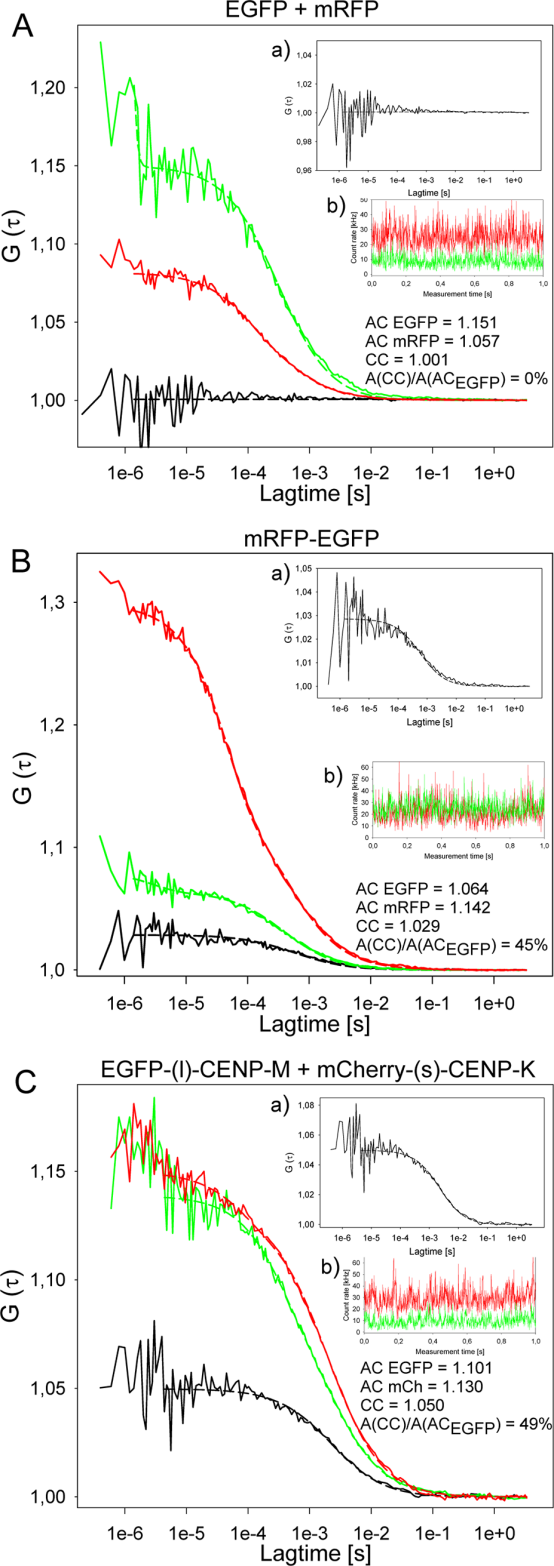
DC-FCCS measurements. Displayed are G versus lag time. Red: FCS- or autocorrelation-curve G (τ) for mRFP (A,B) or mCherry (C), green: FCS- or autocorrelation-curve G (τ) for EGFP, black: cross-correlation-curve G (τ), AC = autocorrelation. CC = cross-correlation, A(AC) = amplitude of autocorrelation curve, A(CC) = amplitude of cross-correlation curve. The cross-correlation analyses are amplified in inserts a. Count rates are displayed over 1 sec (inserts b; green = EGFP and red = mCherry or mRFP) indicating the absence of larger protein aggregates. (A) EGFP and mRFP expressed as single non-fused proteins (negative control) do not show any cross-correlation (A(CC)/A(AC_EGFP_) = 0%). The autocorrelations yielded 1.151 and 1.057 for EGFP and mRFP, respectively. The cross-correlation curve (with a magnified scale of G (τ), insert a) resulted in a value of 1.001 indicating the absence of any complexation between EGFP and mRFP. (B) mRFP-EGFP fusion protein (positive control) shows cross-correlation (A(CC)/A(AC_EGFP_) = 45%). An AC EGFP = 1.064 and an AC mRFP = 1.142 were detected. Cross-correlating the two channels against each other, we obtained a value of 1.029 indicating that about 45% of the molecules are detected as a complex (with a magnified scale of G (τ) in insert a). C) EGFP-(l)-CENP-M and mCherry-(s)-CENP-K indicate complex formation in the nucleoplasm (A(CC)/A(AC_EGFP_) = 49%). The cross-correlation analysis (with a magnified scale of G (τ); insert a) resulted in a correlation of 1.050, whereas the autocorrelations yielded 1.101 and 1.130 for EGFP-CENP-M and mCherry-CENP-K, respectively. The amplitude of the cross-correlation curve A(CC), relative to the diffusion-related amplitude of one of the autocorrelation curves A(AC) of EGFP or mCherry (in this case A(AC_EGFP_), is a measure of binding or dynamic colocalization. According to this ratio of amplitudes, up to 49% of nucleoplasmic CENP-M and -K are part of a common (hetero-dimer or larger) complex.

Tagged proteins were introduced by transient transfection and exhibited centromeric targeting independently of the N- or C-terminal site of fluorescent protein fusion. Before data acquisition, the selected focal volume was bleached, destroying the fluorescence of immobile fluorophores. Thus, after bleaching, only mobile fluorophores (like those tagged to CENPs) can contribute to the DC-FCCS signal. Furthermore, cross talk was minimized by microscope adjustment. Experiments with control fluorochromes in monomeric (EGFP and mRFP) or fused (mRFP-EGFP) forms determined the dynamic range of this system. Co-diffusion generally was detected as described in detail by Bacia & Schwille [120, 121]. First we analyzed EGFP and mRFP as single molecules (negative control, Fig 1A). The count rates of EGFP and mRFP over time, reflecting the fluctuations of the analyzed fluorescent molecules, are displayed in insert b). With the measured signals, autocorrelation analyses were performed (measured signals are compared with itself at later time points and analyzed for repeated motifs) resulting in the FCS- or autocorrelation-curves G (τ) for EGFP (green) and mRFP (red) with calculated autocorrelations (AC) for EGFP (AC EGFP = 1.151) and mRFP (AC mRFP = 1.057). By subtraction of 1 from the AC values, the corresponding amplitudes of the autocorrelation curves A(AC_EGFP_) = 0.151 and A(AC_mRFP_) = 0.057 are obtained. In addition, cross-correlation analyses were performed (measured signals of the green channel were compared with measured signals of the red channel at identical time points and analyzed for identic motifs), resulting in the FCCS- or cross-correlation-curve G (τ) (black). In this example, the calculated cross-correlation (CC) of 1.001 and the amplitude A(CC) = 0.001 indicate 0% co-migration of EGFP with mRFP. In all experiments with this pair of single proteins, the measured cross-correlation values never indicated co-diffusion. For the mRFP-EGFP fusion protein (positive control, Fig 1B) we observed a CC = 1.029, an AC EGFP = 1.064 and an AC mRFP = 1.142. The corresponding amplitudes of the autocorrelation and cross-correlation curves are A(AC_EGFP_) = 0.064, A(AC_mRFP_) = 0.142. and A(CC) = 0.029. A(CC), relative to the diffusion related amplitude of one of the autocorrelation curves (A(AC_EGFP_) in this case), is a measure of binding or dynamic co-localization indicating 45% co-migration. The A(AC)s correspond to the reciprocals of the average numbers of particles in the detection volume, resulting here in 15.6 particles EGFP (N_EGFP_) and 7 particles mCherry (N_mCherry_). The A(CC), however, is directly proportional to the concentration of the co-migrating molecules (N_EGFP-mCherry_). The N_EGFP-mCherry_ can be calculated according to the ratio A(CC)/A(AC_mCherry_) = N_EGFP-mCherry_/N_EGFP_ resulting in about 3.2 co-migrating molecules ((N_EGFP-mCherry_ = 3.2). In addition, N_EGFP-mCherry_ (= 3.2) relative to the number of analyzed molecules of one of the two fluorophores (in this case N_mCherry_ = 7) also indicated 45% co-migration. In general, we obtained CC-values between 40 and 55% for mRFP-EGFP and EGFP-mCherry fusion proteins [90,97]. These results are in agreement with results of Kohl & Schwille [130]. For such fusion proteins, 100% cross-correlation should be observed. Since cross-correlation values obtained with these protein pairs rate the factual percentage of co-migrating molecules too low by approximately a factor of two, we corrected the values measured with DC-FCCS by this factor. The lower values of 40–55% is due to at least four effects [124]: (i) differences in observation volumes for fluorescent proteins of different emission wavelengths and displacement of these observation volumes due to chromatic aberrations, (ii) the existence of dark fluorescent proteins due to maturation problems [130] and photo-bleaching, (iii) Förster resonance energy transfer (FRET) between the labels changing the amplitudes of auto- and cross-correlations, and finally (iv), endogenous proteins might interfere with the analysed biomolecular interactions. The dominant reason seems to be that roughly only about half of all mCherry fluoresce (see Materials & Methods). Thus, cross-correlation values obtained from DC-FCCS experiments under-estimate the percentage of co-migrating molecules. In order to avoid these effects, we carry out single wavelength fluorescence cross-correlation (SW-FCCS) experiments (see below). In addition, the fluorescent tag itself, in particular when being tied by a short linker, might interfere with protein-protein interaction, for example by being attached at or close to the binding site, introducing sterical hindrance. Therefore, when detecting no protein-protein interaction, in most cases additional cross-correlation experiments were carried out with (i) proteins labeled at the other terminus and/or (ii) introducing a long protein linker between EGFP and CENP, reducing or excluding the effects of sterical hindrance by the tagged fluorescent protein.

### CENP-H/I/K/M complex

As a further CCAN sub-complex next to CENP-P/O/R/Q/U [90], we analysed the potential pairwise interactions between proteins of the CENP-H/I/K/M sub-complex [35,92] in order to determine if CENP-H/I/K/M proteins form hetero-dimers or -multimers in the nucleoplasm outside centromeres. By Y2H we only detected a single weak interaction between CENP-H and -K. In double-transfected interphase HEp-2 cells, we analysed various combinations of EGFP and mCherry tagged CENP-H/I/K/M proteins (Table 1). Measurements of EGFP-(l)-CENP-M and mCherry-(s)-CENP-K in the nucleoplasm (CENP-M was fused to EGFP via a long linker, whereas CENP-K and mCherry were connected by a short linker, see Materials & Methods) showed individual autocorrelation values of 1.101 (EGFP) and 1.130 (mCherry). Cross-correlation measurements resulted in a CC = 1.050, indicating that in this cell 49% of the molecules were co-migrating (Fig 1C, Table 1). In total, from 21 FCCS measurements carried out in the nucleoplasm of 13 cells, 19 measurements in 12 cells indicated that outside kinetochores (37±9)% of EGFP-(l)-CENP-M and mCherry-(s)-CENP-K were co-diffusing and thus co-resident in a single complex. Since cross-correlation values under-estimate the percentage of co-migrating molecules (see above) roughly by the factor two, hetero-dimerisation of EGFP-(l)-CENP-M and mCherry-(s)-CENP-K is expected to be twofold higher than the calculated (37±9)%; we estimate a corrected value of about 56–92%. When measuring the cross-correlation between EGFP-(s)-CENP-I and mCherry-(s)-CENP-M as well as between CENP-I-(s)-EGFP and CENP-M-(s)-mCherry (all fusions with short linkers), we found no cross-correlation (Table 1). We speculated that the fluorescent tag might interfere with protein-protein interaction (see above). In order to test this hypothesis, we introduced a long protein linker between EGFP and CENP-M and repeated the cross-correlation experiment, now between mCherry-(s)-CENP-I and EGFP-(l)-CENP-M. In this case, when EGFP is fused to CENP-M by a long linker, we found (24±9)% of the molecules co-migrating (corrected value 30–66%) (Table 1). In further experiments we measured the cross-correlation, and found co-migration, between EGFP-(s)-CENP-H and CENP-I-(s)-mCherry, between EGFP-(s)-CENP-H and mCherry-(s)-CENP-K and between EGFP-(s)-CENP-K and mCherry-(s)-CENP-I (Table 1). By FCCS in the nucleoplasm outside centromeres, we estimate pairwise co-migration of CENP-H/I/K/M proteins in the range 30 to >90% (corrected values, considering the under-estimation discussed above; see Fig 1B and 1C). This suggests that in the nucleoplasm of interphase cells outside centromeres, most of the CENP-H/I/K/M proteins (30 to >90%) form a multimeric complex.

Our results are in agreement with earlier observations. CENP-H [67,131,132] and CENP-K [35,68,100,133] form a tight dimer when co-expressed in and purified from insect cells [92]. CENP-H and CENP-K interact with CENP-I [35,92,101,134] and were proposed to form a complex [31,32,35]. CENP-M stabilizes this complex [92]: CENP-I bridges CENP-H/K and CENP-M; both CENP-H/K and CENP-M contribute to CENP-I stabilisation. This stabilisation is functionally relevant since CENP-M and CENP-I are mutually required for kinetochore localization. Thus, these proteins form a quaternary complex [12,13] which, as indicated by our data here, partially forms already in the nucleoplasm outside centromeres (see Fig 5).

### CENP-C

CENP-C is required for the incorporation and stabilisation of CENP-A nucleosomes [63,81], has a central CCAN forming function [12] and recruits the CENP-T/W/S/X and CENP-H/I/K/M complexes to centromeres. CENP-C binds the CENP-L/N and CENP-H/I/K/M complexes directly, with CENP-H and CENP-K being the main determinants of this interaction [12–15,92]. By DC-FCCS we therefore measured co-migration between EGFP-(s)-CENP-H and CENP-C tagged at either end (mCherry-(s)-CENP-C and CENP-C-(s)-mCherry). We found co-migration between CENP-H and CENP-C in the range of 16 to 66% (corrected, Table 1). This indicates that in the nucleoplasm outside centromeres, CENP-C interacts with CENP-H (either directly or indirectly), and suggests that there CENP-C is associated with the CENP-H/I/K/M complex, consistent with [15] (see Fig 5).

### CENP-N/L complex

Next we studied the CENP-N/L complex. CENP-N associates with CENP-A nucleosomes and directly binds CENP-L [12,15,75,79,80]. By DC-FCCS we identified a cross-correlation between EGFP-(l)-CENP-L and mCherry-(s)-CENP-N ((23±9)% co-migration, corrected value 28–64%) (Table 1), indicating that most of the CENP-N and CENP-L proteins co-migrate. Thus, our data show that to a considerable amount CENP-N and CENP-L already interact in the nucleoplasm outside centromeres. CENP-K and CENP-N interact with one another [75], probably inducing an interaction between the complexes CENP-L/N and CENP-H/I/K/M [12,15]. We therefore measured DC-FCCS between EGFP-(l)-CENP-L and mCherry-(s)-CENP-K and between CENP-K-(s)-EGFP and mCherry-(s)-CENP-N, and found co-migration between 26 and 78% (corrected, see Table 1). The results indicate that in the nucleoplasm about one to two thirds of the available CENP-N and CENP-L proteins (most of them complexed together, see above) associated with proteins of the CENP-H/I/K/M complex. This is consistent with the recent observation that CENP-N seems to be associated with CENP-H/I/K/M, however not always [96]. Interestingly, DC-FCCS between EGFP-(s)-CENP-N and mCherry-(s)-CENP-K showed no co-migration (Table 1). The CENP-K N-terminal, but not the C-terminal tag, fused by a short linker, seems to interfere with binding to CENP-N. In summary, in the nucleoplasm outside centromeres, the CENP-L/N complex is partly associated with the CENP-H/I/K/M complex (see Fig 5).

### CENP-T/W/S/X complex

We then studied the interactions between CENP-T/W/S/X proteins [91] in the nucleoplasm not only by DC-FCCS (as above) but also in more detail by quantitative SW-FCCS [124,135–137]. This approach allows the estimation of effective dissociation constants. In principle, the ratio of the auto-(ACF) and cross-(CCF) correlation amplitudes can vary between zero (no binding) and one (complete binding (assuming 1:1 stoichiometry)). Factors affecting the correlation amplitudes and their ratios include background fluorescence (which in our DC-FCCS experiments was eliminated by bleaching the focal volume before data acquisition) and cross talk [138–140] (which was minimized by microscope adjustment). Nevertheless, the largest values for the CCF/ACF amplitude ratios for fused fluorescent proteins (like EGFP-mRFP or EGFP-mCherry) were in the range of ~0.5 (see Fig 1, Table 1), due to differences in observation volumes for different fluorescent proteins, the existence of dark fluorescent proteins and photo-bleaching, Förster resonance energy transfer (FRET), and endogenous protein interference with the analysed biomolecular interactions (see above). Recently, we established procedures for correcting these effects [124].

Single wavelength FCCS (SW-FCCS) overcomes the difficulty of aligning two lasers to the same spot [135–137]. Further quantitative corrections allowed us to accurately determine the ACF and CCF amplitudes and thus obtain correct concentrations of the interacting molecules so that we can deduce K_d_ values. K_d_ values from *in vivo* experiments usually are effective dissociation constants since in many cases interactions in cells are not simple pure binary reactions but can be affected by the presence of other biomolecules. Even unspecific background reactions of low binding strength can influence the measured K_d_ if the concentration of the unspecific binding partner is high. Nevertheless, in the absence of detailed knowledge of the composition and all interactions in a cell, this effective K_d_ is a relevant parameter.

First, by SW-FCCS we analyzed mCherry and EGFP expressed in interphase HEK293 cells as single proteins (negative control) or expressed as the fused hetero-dimer mCherry-EGFP (positive control). The diffusion time τ_D_ of mCherry-EGFP was slightly higher than those of the single proteins (Table 2). The uncorrected amount of co-migration was (8.6±6.7)% for the single proteins (defining the background level) and (47±14)% for the hetero-dimer (Table 2), defining the maximally expected value.

**Table 2:** Cross-correlation by SW-FCCS.

Sample	τ_D_ ± SD (ms)	Dimer ±	Measurements /	*K*_*d*_ ± SD	Corrected *K*_*d*_ ± SD	Corrected
		SD (%)	number of cells			Dimer ± SD (%)
Control measurements						
mCherry-EGFP	G: 0.87 ± 0.16	47.4 ± 14.0	37 / 17	N/A	N/A	N/A
	R: 0.95 ± 0.22					
EGFP	G: 0.65 ± 0.45	8.6 ± 6.7	12 / 8	N/A	N/A	N/A
mCherra	R: 0.60 ± 0.09					
Interacting proteins^1^						
EGFP-(s)-CENP-S	G: 2.86 ± 2.02	41.6 ± 12.0	101 / 36	409 nM ±28 (S)	264 nM ± 37 (S)	58 ± 15
mCherry-(s)-CENP-X	R: 2.23 ± 1.53			679 nM SD_ln_ 1.6 (H)	287 nM SD_ln_ 0.5 (H)	
EGFP-(s)-CENP-W	G: 6.41 ± 4.40	35.4 ± 14.9	218 / 87	1.5 μM ± 0.1 (S)	538 nM ± 81 (S)	51 ± 18
mCherry-(s)-CENP-T	R: 5.61 ± 5.53			626 nM SD_ln_ 1.7 (H)	340 nM SD_ln_ 1.2 (H)	
EGFP-(s)-CENP-T^∆N^	G: 3.6 ± 2.3	18.2 ± 15.2	79 / 16	2.4 μM ± 0.2 (S)	1.3 μM ± 0.2 (S)	24 ± 17
mCherry-(s)-CENP-W	R: 2.7 ± 1.7			1.3 μM SD_ln_ 1.5 (H)	944 nM SD_ln_ 1.9 (H)	
Non-interacting proteins^2^						
EGFP-(s)-CENP-W	G: 1.6 ± 0.8	6.1 ± 5.5	11 / 5	N/A	N/A	N/A
mCherry-(s)-CENP-X	R: 1.5 ± 0.7					
EGFP-(s)-CENP-S	G: 3.3 ± 2.8	11.5 ± 7.8	12 / 6	N/A	N/A	N/A
mCherry-(s)-CENP-W	R: 1.4 ± 1.0					
EGFP-(s)-CENP-T^∆C^	G: 7.0 ± 2.5	10.9 ± 8.1	23 / 7	N/A	N/A	N/A
mCherry-(s)-CENP-W	R: 1.4 ± 3.2					
EGFP-(s)-Spc24	G: 10.6 ± 7.5	10.3 ± 11.6	14 / 4	N/A	N/A	N/A
mCherry-(s)-CENPT^∆N^	R: 5.3 ± 2.6					
Possibly weakly interacting proteins^3^						
mCherry-(s)-CENP-T	G: 2.4 ± 1.9	12.0 ± 8.9	7 / 5	5.8 μM ± 0.2 (S)	N/A	N/A
EGFP-(s)-CENP-X	R: 2.9 ± 1.2					
EGFP-(s)-Spc24	G: 11.8 ± 17.3	13 ± 13	29 / 8	1.8 μM ± 0.2 (S)	N/A	N/A
mCherry-(s)-CENP-T	R: 8.1 ± 4.7					
EGFP-(s)-CENP-S	G: 2.04 ± 1.54	10.5 ± 8.6	61 / 28	9.3 μM ± 2.3 (S)	N/A	N/A
mCherry-(s)-CENP-T	R: 3.8 ± 1.20			12.0 μM SD_ln_ 1.6 (H)		
EGFP-(s)-Spc25	G: 9.0 ± 7.7	10.3 ± 7.4	15 / 4	N/A	N/A	N/A
mCherry-(s)-CENP-T^∆C^	R: 7.0 ± 9.9					

1. The interacting proteins have both, a) a cross-correlation above the negative control and, b) similar diffusion coefficients for red- and green-labeled molecules.

2. The non-interacting proteins have a) cross-correlations close to or at the negative control and b) different diffusion coefficients for the red- and green-labeled molecules.

3. The proteins fulfill both conditions (as in 1), they have a) cross-correlation above the negative control and, b) similar diffusion coefficients for red- and green-labeled molecules. However, their cross-correlation is only slightly different from the negative control and thus their interaction cannot be ascertained under the measurement conditions. The original data behind “means” and “SD” are listed in S2 Table.

(S) = scatter plot

(H) = histogramm

**Fig 2:**
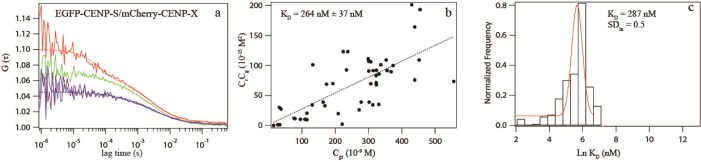
SW-FCCS analysis of CENP-S and CENP-X. (a): ACF curves of EGFP-(s)-CENP-S (green) and mCherry-(s)-CENP-X (red), and CCF curves (blue, purple) in the nucleoplasm of interphase HEK293 cells. The data show high cross-correlation between CENP-S and CENP-X, indicating an interaction between CENP-S and CENP-X. (b) and (c): *K*_*d*_ determination using Scatter plot (b) and a histogram (c) of multiple SW-FCCS measurements to determine the effective *K*_*d*_ of the interaction.

Recently, by DC-FCCS we detected a cross-correlation between EGFP-(s)-CENP-X and mCherry-(s)-CENP-S in the nucleoplasm, suggesting that 60–100% (corrected value) of the molecules co-migrate and thus co-reside in a single complex (Table 1) [97]. By contrast, the FCCS analysis of CENP-S association with CENP-T revealed no detectable soluble complex containing these two proteins, consistent with our Y2H experiments which also did not detect an interaction between CENP-T and CENP-S. We concluded that, outside centromeres and ectopic chromatin sites, to a large extent CENP-S and CENP-X exist in a diffusible complex independent of CENP-T [97]. Here, we repeated this experiment, now fusing mCherry to CENP-S by a long linker. By DC-FCCS we measured a cross-correlation between EGFP-(s)-CENP-X and mCherry-(l)-CENP-S ((31±7)% co-migration, corrected value 48–76%) (Table 1), confirming our previous results. Then, we exchanged the N-terminal tags and studied EGFP-(s)-CENP-S and mCherry-(s)-CENP-X by SW-FCCS. In 101 measurements in 36 cells, both tagged proteins showed similar diffusion times and high uncorrected co-migration of (42±12)% (Table 2). The fusion proteins moved slower than the single fluorescent proteins. From our data we deduced an effective dissociation constant K_d_ of (264±37) nM from the scatter plot and the similar value 287 nM (SD_ln_ 0.5) from the histogram analysis (Fig 2, Table 2). When correcting these data according to [124], we obtained (58±15)% as the relative amount of complex formation (Table 2). Thus, in agreement with our DC-FCCS results, most of the CENP-S and CENP-X molecules form a complex in the nucleoplasm of interphase cells outside centromeres.

*In vitro*, in the absence of CENP-T/W, CENP-S/X can assemble into a tetramer [91]. CENP-T/W or CENP-S/X complexes alone can induce negative supercoils into DNA similar to canonical histones, while the CENP-T/W/S/X complex induces positive supercoils [98]. Here we observed that the diffusion times of EGFP-(s)-CENP-X and mCherry-(s)-CENP-S are slower than those of the single proteins mRFP and EGFP but clearly faster than the diffusion time of mCherry-H4. Potentially, this suggests that in the nucleoplasm EGFP-(s)-CENP-X and mCherry-(s)-CENP-S are not or only to a small extent incorporated into chromatin structures. A freely diffusible CENP-S/X complex would support its non-centromeric functional activities at DNA repair sites [141,142].

**Fig 3:**
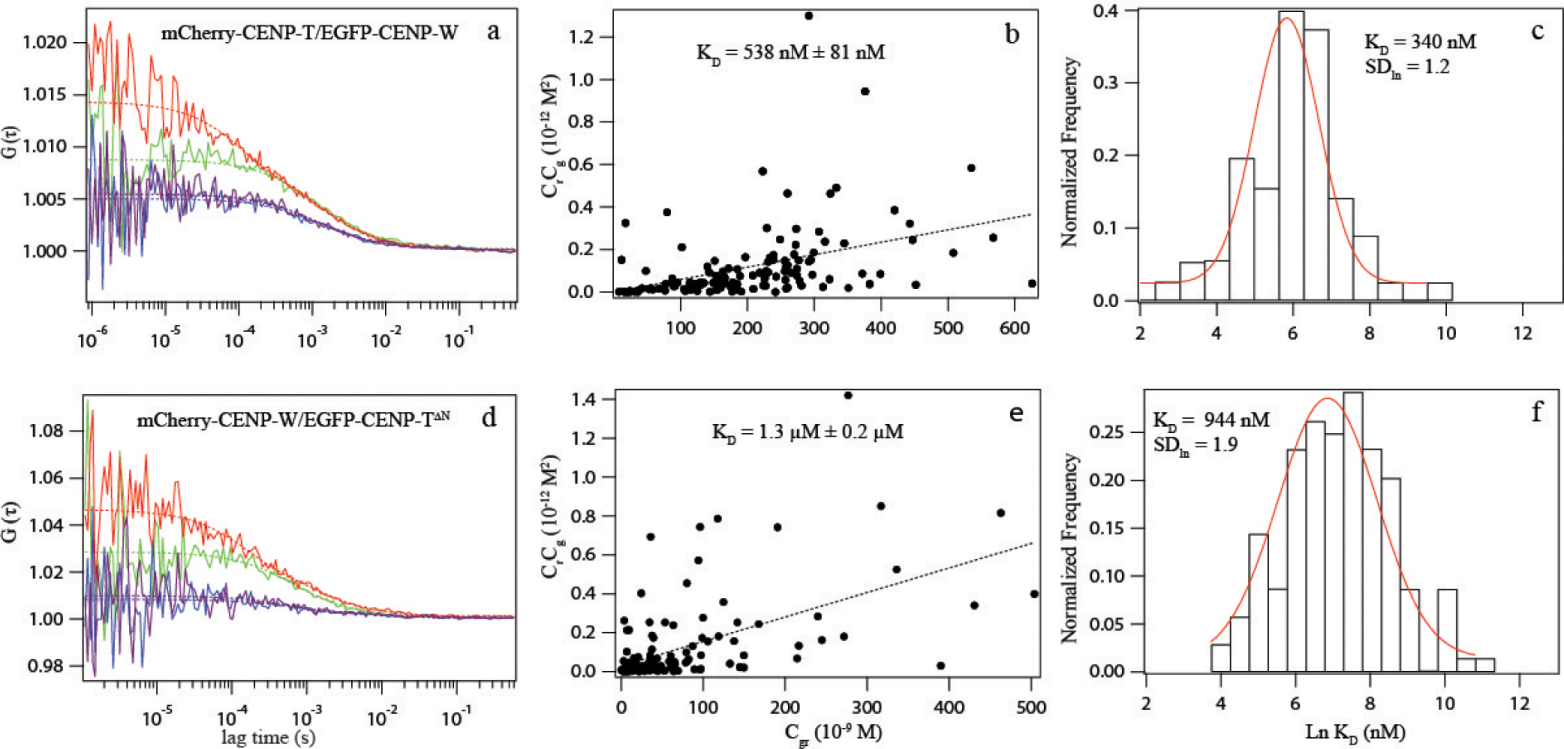
SW-FCCS analysis of CENP-T and CENP-W. (a): ACF curves of EGFP-(s)-CENP-W (green) and mCherry-(s)-CENP-T (red), and CCF curves (blue, purple) in the nucleoplasm of interphase HEK293 cells. The data show cross-correlation between CENP-T and CENP-W, indicating interaction. (b) and (c): *K*_*d*_ determination using Scatter plot (b) and a histogram (c) of multiple SW-FCCS measurements to determine an effective *K*_*d*_ of the interaction. (d): ACF curves of EGFP-(s)-CENP-T^∆N^ (green) and mCherry-(s)-CENP-W (red), and CCF curves (blue, purple) in the nucleoplasm of interphase HEK293 cells. The data show reduced cross-correlation between CENP-T^∆N^ and CENP-W. (e) and (f): *K*_*d*_ determination using Scatter plot (e) and a histogram (f) of multiple SW-FCCS measurements to determine the effective *K*_*d*_ of this interaction between the histone-fold domain of CENP-T (CENP-T^∆N^) and CENP-W. A defined interaction is detected by both, the linear fit of the scatter plot as well as the log-normal fit of the histogram.

Then we studied the interaction between CENP-T and CENP-W [70,91]. By DC-FCCS, we measured a cross-correlation between CENP-T-(s)-EGFP and mCherry-(s)-CENP-W ((28±8)% co-migration, corrected value 40–72%) (Table 1). We switched the tags and repeated the analysis by SW-FCCS. In 218 measurements in 87 cells, EGFP-(s)-CENP-W and mCherry-(s)-CENP-T showed similar mobility in the nucleoplasm (slightly slower than the CENP-S and CENP-X fusion proteins) and high uncorrected co-migration (35±15)% (Table 2). Correcting these values, an effective dissociation constant K_d_ of (538±81) nM was deduced from the scatter plot and of 340 nM (SD_ln_ 1.2) from the histogram analysis (Fig 3). The corrected value for the relative amount of complex formation was (51±18)% (Table 2), consistent with our DC-FCCS results.

CENP-T binds DNA *in vitro* and *in vivo* [70,98]. Thus, in the chromatin environment of the nucleoplasm, single CENP-T and CENP-T/W complexes envisage multiple DNA binding sites. This disturbs our binding analysis and complicates the interpretation of the measured effective K_d_ for the interaction between CENP-T and CENP-W. We therefore constructed two separate CENP-T domains: the CENP-T mutants CENP-T^∆C^ (aas 1–395) and CENP-T^∆N^ (aas 396–561). We found no co-migration between the N-terminal domain of CENP-T (EGFP-(s)-CENP-T^1-395^) and CENP-W (mCherry-(s)-CENP-W) (Table 2), indicating that the C-terminal domain CENP-T^396-561^, containing the histone-fold domain (aas 376–561), interacts with CENP-W, supporting results by [70]. Next, by SW-FCCS we analysed the interaction between the C-terminal domain of CENP-T (EGFP-(s)-CENP-T^396-561^) and mCherry-(s)-CENP-W. We found similar mobility in the nucleoplasm and clear co-migration of (24±17)% (corrected value) with an effective K_d_ of (1.3±0.2) μM (scatter plot) and 944 nM (SD_ln_ 1.9) (histogram) (corrected values, Fig 3, Table 2). Compared to wildtype, we thus found modified binding between CENP-W and CENP-T^∆N^, with a shifted effective K_d_. Taken together, for both complexes, CENP-T/W and CENP-S/X, in the nucleoplasm we measured apparent dissociation constants in the 1 μM range. At the centromere, we expect stronger binding to the kinetochore multi-protein complex due to additional protein-protein contacts by CENP proteins.

**Fig 4:**
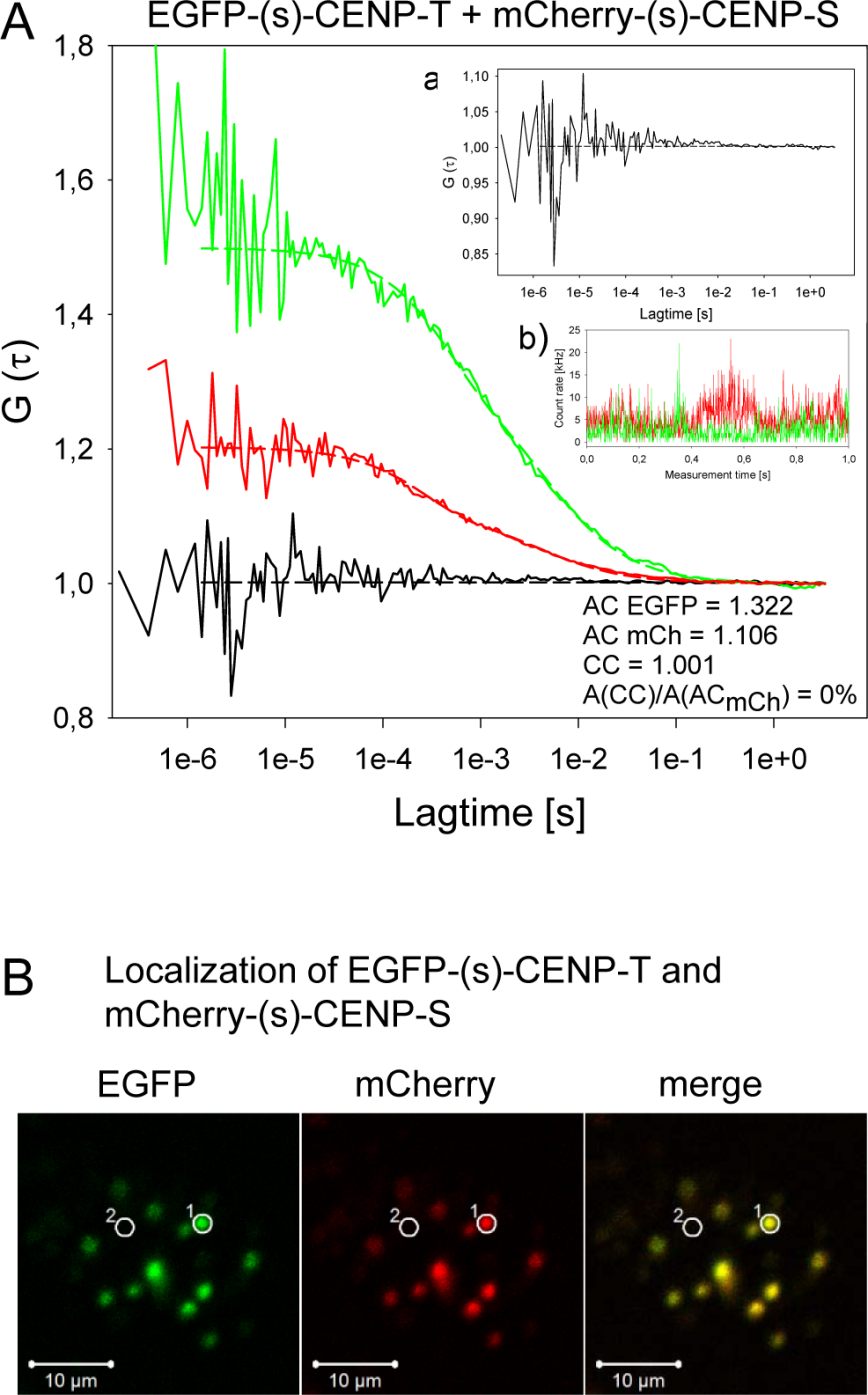
DC-FCCS of EGFP-(s)-CENP-T and mCherry-(s)-CENP-S. A) Displayed are G versus lag time. Red: FCS- or autocorrelation-curve G (τ) for mCherry, green: FCS- or autocorrelation-curve G (τ) for EGFP, black: cross-correlation-curve G (τ), AC = autocorrelation. CC = cross-correlation, A(AC) = amplitude of autocorrelation curve, A(CC) = amplitude of cross-correlation curve. The cross-correlation analyses are amplified in inserts a. Count rates are displayed over 1 sec (inserts b; green = EGFP and red = mCherry). For the pair EGFP-(s)-CENP-T and mCherry-(s)-CENP-S no indication for complex formation in the nucleoplasm was detected (A(CC)/A(AC_mCherry_) = 0%). The cross-correlation analysis (with a magnified scale of G (τ); insert a) resulted in a correlation of 1.001, whereas the autocorrelations yielded 1.322 for EGFP-(s)-CENP-T and 1.106 for mCherry-(s)-CENP-S. This ratio indicates that no nucleoplasmic CENP-T and -S are part of a common complex. B) Localization of cotransfected EGFP-(s)-CENP-T (EGFP) and mCherry-(s)-CENP-S (mCherry) in living human HEp-2 cells which were used for FCCS analysis. White bar = 10 μm. A cell nucleus is displayed showing co-localisation at centromeres (merge) and weak fluorescence in the nucleoplasm. Two locations of the same size and shape, a centromere (spot 1) and the centromere-free position of an FCCS measurement, as shown in Fig 4A (spot 2), in the nucleoplasm were selected for fluorescence intensity analysis. For the analyzed centromere in spot 1 the ratios of nucleoplasmic to centromeric fluorescence intensities was 1:43 for EGFP-(s)-CENP-T and 1:33 for mCherry-(s)-CENP-S. The concentrations of nucleoplasmic proteins, estimated by FCCS, was 6 nM for EGFP-(s)-CENP-T and 14 nM for mCherry-(s)-CENP-S.

By DC-FCCS, in the nucleoplasm, we found no cross-correlation between EGFP-(s)-CENP-T and mCherry-(s)-CENP-S, between EGFP-(s)-CENP-T and CENP-S-(s)-mCherry, between EGFP-(s)-CENP-T and mCherry-(l)-CENP-S and between EGFP-(l)-CENP-T and mCherry-(l)-CENP-S (Table 1), independent of the linker lengths of the fusion proteins. Analysis of EGFP-(s)-CENP-T and mCherry-(s)-CENP-S in the nucleoplasm showed autocorrelation values AC EGFP = 1.322 and AC mCherry = 1.106. Cross-correlation measurements resulted in a CC = 1.001, indicating that the molecules were not co-migrating (Fig 4A, Table 1). The localization of EGFP-(s)-CENP-T and mCherry-(s)-CENP-S in the nucleus of a living human HEp-2 cells, used for FCCS analysis, is shown in Fig 4B. Compared to centromeres, the fluorescence intensity in the nucleoplasm is rather weak. For the analyzed centromere in spot 1 the ratios of nucleoplasmic to centromeric fluorescence intensities was 1:43 for EGFP-(s)-CENP-T and 1:33 for mCherry-(s)-CENP-S. A(AC_EGFP_) = 0.322 and A(AC_mCherry_) = 0.106 correspond to the reciprocals of the average numbers of particles in the detection volumes. Accordingly 3.1 particles EGFP-(s)-CENP-T and 9.1 particles mCherry-(s)-CENP-S were found in the analyzed confocal detection volume. When knowing the effective detection volume, the concentrations of the samples can be deduced. Rüttinger et al. [143] described a linear dependence of the number of particles detected by FCS with the concentrations of the analyzed samples in a range between 0.5–100 nM. From these results, under their experimental conditions, they determined an effective detection volume of (1.0±0.1) fl for Atto-655 in H_2_O. Under our experimental conditions, with a purified EGFP solution with known concentration, for EGFP we determined an effective detection volume of roughly 0.8 fl. Due to the above described problems with insufficient maturation and stability of mRFP and mCherry, we could not experimentally determine the effective detection volume for these proteins. Due to the longer emission wavelengths we estimated them to be about 30% larger than the detection volume of EGFP. Thus, for calculations with mCherry we used an effective detection volume of 1.04 fl. Determining the effective detection volume of the fusion proteins in living cells is more complex due to different local compositions and viscosity, and additional interacting components, influencing the mobility of the proteins. Therefore, for DC-FCCS experiments the intracellular concentrations were only estimated. Using the effective detections volumes measured in solution (see above), nucleoplasmic protein concentrations were deduced to be 6 nM for EGFP-(s)-CENP-T and 14 nM for mCherry-(s)-CENP-S.

**Fig 5:**
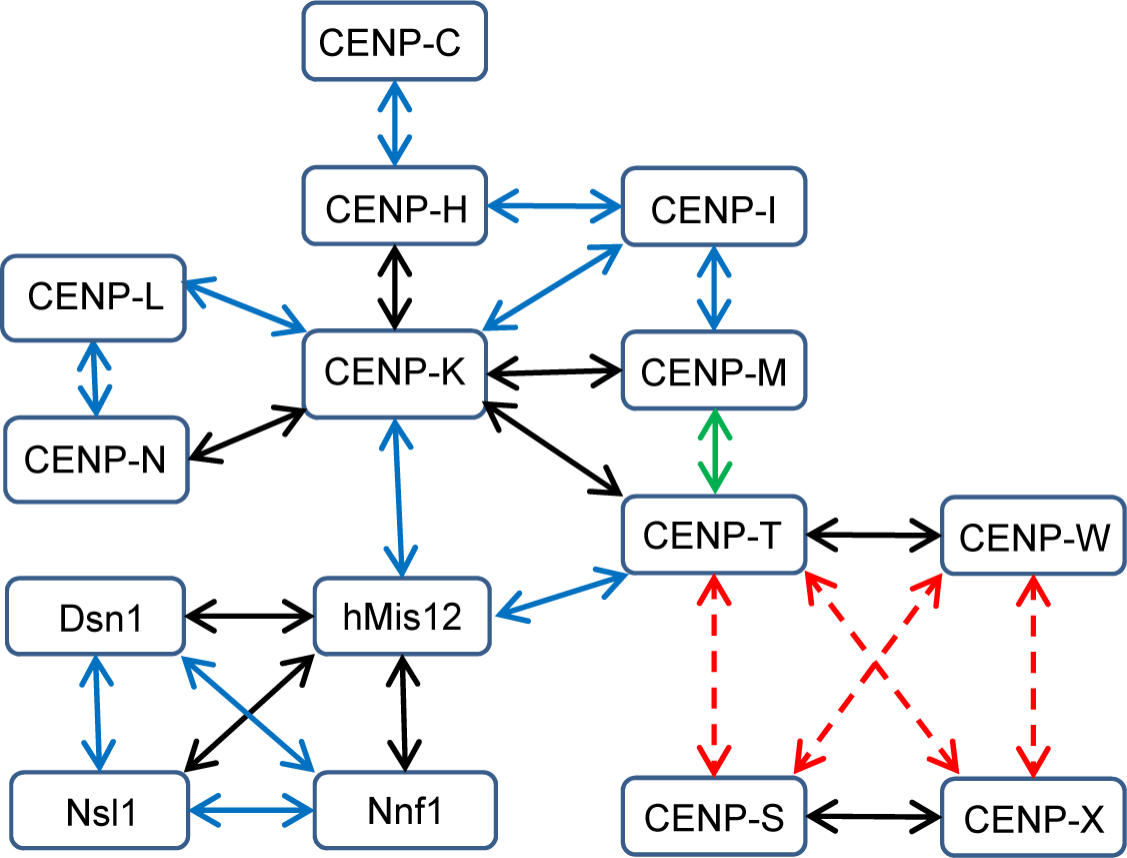
Protein-protein co-migration of CCAN and Mis12 proteins. The degree of co-migration (corrected) in the nucleoplasm outside centromeres of human interphase cells is color-coded (red dashed arrows: no or hardly detectable co-migration, green arrow: 5–30%, blue arrows: 30–60%, black arrows: above 60% co-migration). Please note: these green, blue or black arrows do not necessarily indicate direct protein-protein interaction; the labeled proteins co-migrate in a complex: their interaction might be either direct or mediated by a third (or more) protein.

We neither found co-migration between EGFP-(s)-CENP-T and mCherry-(s)-CENP-X nor between EGFP-(l)-CENP-W and CENP-X-(l)-mCherry (Table 1). Using different linker lengths and a switched tag for CENP-X, we confirmed the latter result by SW-FCCS. Furthermore, we found no co-migration between EGFP-(s)-CENP-S and mCherry-(s)-CENP-W (Table 2). In SW-FCCS, for mCherry-(s)-CENP-T/EGFP-(s)-CENP-X and EGFP-(s)-CENP-S/mCherry-(s)-CENP-T the cross-correlations are slightly above the negative control value, and the diffusion times in both channels are close (Table 2). According to these data, we cannot rule out possibly very weak interactions between these two protein pairs in the nucleoplasm. In summary, our results indicate that, consistent with earlier observations [23,89,91], CENP-T and CENP-W as well as CENP-S and CENP-X form two separate hetero-dimeric complexes which, in the nucleoplasm outside centromeres, practically do not co-migrate as a joint larger complex (see Fig 5).

Human CENP-T binds to Spc24/25 of the human Ndc80 complex [21–24,84]. This interaction is influenced by CENP-T phosphorylation [21,22]: CENP-T is phosphorylated on multiple cyclin dependent kinase (CDK) consensus sites in G2 of HeLa cells and remains phosphorylated until anaphase. *In vivo*, CENP-T phosphorylation is important since its elimination prevents Ndc80 localization and causes defects in chromosome segregation [83]. However, also the un-phosphorylated human CENP-T^76-106^ N-terminal fragment binds to the human Spc24^137–197^ /Spc25^129–224^ complex (with a K_d_ of 645 nM) while phospho-mimetic human CENP-T shows an increased K_d_ of 150 nM [84]. In the nucleoplasm of interphase cells, by SW-FCCS we analysed EGFP-(s)-Spc24 and mCherry-(s)-CENP-T and detected a very weak cross-correlation; we deduced a K_d_ of 1.8 μM (Table 2). EGFP-(s)-Spc25 and mCherry-(s)-CENP-T^1-395^ showed an even weaker cross-correlation with a K_d_ of (3.0±0.9) μM (scatter plot) and 4.3 μM (SD_ln_ 0.9) (Table 2). These values are consistent with *in vitro* results [24,84]. That these weak cross-correlations slightly above background level might indeed indicate co-migration and complex formation is supported by our observation that in both cases the fusion proteins move with similar diffusion times.

CENP-T/W interacts with the CENP-H/I/K/M complex, an interaction mediated by CENP-H/K [12,13,15,21,23,70,92]. *In vitro*, CENP-T/W was found to bind CENP-K [83]. By DC-FCCS we studied CENP-K-(s)-EGFP and CENP-T-(s)-mCherry and found (31±13)% co-migration (corrected value 36–88%). Also for EGFP-(s)-CENP-M and CENP-T-(s)-mCherry we measured 20–32% (corrected value) co-migration (Table 1). A co-migration with CENP-M was not detected when replacing full-length CENP-T by its N-terminal domain CENP-T^∆C^ (CENP-T^1-395^-(s)-mCherry). Thus, the co-migration of CENP-T with CENP-M is caused by its C-terminal domain (in agreement with yeast data [23]). Our data suggest that, in the nucleoplasm outside kinetochores, the proteins of the CENP-H/I/K/M complex form a complex with the CENP-T/W hetero-complex (see Fig 5). The measured pairwise co-migrations do not necessarily indicate a direct protein-protein interaction between the two analysed proteins: the detected co-migration might be mediated by a third protein (or more) in the nucleoplasm which probably would be a CENP protein. In the nucleoplasm, the hetero-dimer CENP-S/X does not bind to the CENP-C/H/I/K/M/T/W proteins (Fig 5); it becomes part of the CCAN kinetochore complex only at centromeres. Recently we observed by fluorescence-three-hybrid studies (F3H) that *in vivo* CENP-K is able to recruit CENP-O and CENP-U to non-centromeric chromatin sites, and that CENP-L can recruit CENP-R [90]. We thus speculate that at least also CENP-O/U/R of the CENP-P/O/R/Q/U proteins bind to the CENP-C/H/I/K/M/T/W/N/L proteins *in vivo* in the nucleoplasm outside centromeres.

### hMis12 complex

Finally, by DC-FCCS we studied the proteins hMis12, Nsl1, Dsn1 and Nnf1 of the hMis12 complex [77,85,144,145]. We measured the cross-correlation between these four proteins and found co-migration for all six protein pairs in the range between about 20 and 90% (corrected values) (Table 1), indicating that a large fraction of these proteins in the nucleoplasm are part of complexes, either of hetero-dimers or -multimers (Fig 5). Our results are in agreement with published conclusions that the four proteins of the hMis12 complex form a quaternary complex [25,26,144,145]. Here we find that, in the nucleoplasm outside centromeres, a larger part of these proteins aggregate to hetero-dimers and hetero-multimers (Fig 5). *In vitro*, the hMis12 complex directly interacts with the CCAN, in particular with CENP-C, CENP-T, CENP-K and CENP-N [21,22,24,25,106]. Indeed, FRAP showed dynamic binding to centromeres with fast protein exchange in interphase cells [116] indicating weak binding. Here we showed that 30–44% of hMis12 of the Mis12 complex proteins co-migrated with CENP-K and CENP-T *in vivo* in the nucleoplasm outside centromeres (Table 1), indicating interaction of hMis12 (and probably the whole Mis12 complex) with the CENP-C/H/I/K/M/T/W/N/L complex. In mitosis, hMis12 stably binds to centromeres [116]. This stable interaction requires protein phosphorylation which *in vivo* takes place only in mitosis [29].

## Conclusion

In this work, by FCCS we measured the co-migration of CCAN and hMis12 complex protein pairs in the nucleoplasm outside centromeres in living human interphase cells. Since cross-correlation values obtained from DC-FCCS experiments under-estimate the percentage of co-migrating molecules, for some protein pairs we carried out SW-FCCS experiments which correct for these effects. Furthermore, the fluorescent tags might introduce sterical hindrance, preventing the detection of protein-protein interaction. Therefore, we labeled proteins at both termini and, in cases we detected no co-migration, introduced longer protein linkers between tag and CENP. For the hetero-dimerisation of CENP-T/W and CENP-S/X, we also determined apparent dissociation constants (K_d_). We detected co-migration between CENP-K and CENP-T as well as between CENP-M and CENP-T but not between CENP-T/W and CENP-S/X. Furthermore, CENP-C co-migrated with CENP-H, and CENP-K with CENP-N as well as with CENP-L. Thus, in the nucleoplasm outside centromeres, the CENP-H/I/K/M proteins interact with CENP-C, CENP-N/L and CENP-T/W but not with CENP-S/X. Probably these interactions take place in the nucleoplasm independent of modifications specifically occurring at centromeres. Nevertheless, we assume that at the centromere cell cycle and location specific protein modification of at least some of the proteins will enhance their interactions with other CCAN proteins. Furthermore, at the centromere CCAN protein-protein interactions will be further stabilized by multiple protein-protein binding [146]. Our FCCS analysis of the hMis12 complex showed that also these four proteins form a diffusing complex outside centromeres with at least hMis12 interacting with members of the CENP-C/H/I/K/M/T/W/N/L complex. Overall we show here that human CCAN and hMis12 kinetochore proteins assemble in the nucleoplasm outside centromeres, potentially forming dynamic multimeric complexes.

## Supporting information

S1 Table Cross-correlation of protein pairs in the nucleoplasm by DC-FCCS. Original Data from which “means” and “SD” values were calculated and presented in Table 1.
(DOCX)

S2 Table Cross-correlation of protein pairs in the nucleoplasm by SW-FCCS. Original Data from which “means” and “SD” values were calculated and presented in Table 2.
(DOCX)
