# Supplementary material for: CENP-C/H/I/K/M/T/W/N/L and hMis12 but not CENP-S/X participate in complex formation in the nucleoplasm of living human interphase cells outside centromeres
Source: PLoS One. 2018 Mar 6;13(3):e0192572. doi: 10.1371/journal.pone.0192572 (PMC5839545; doi:10.1371/journal.pone.0192572)
Supplement: S1 Table — Original Data from which “means” and “SD” values were calculated and presented in Table 1. (DOCX) [file pone.0192572.s001.docx]

**S1 Table. Cross-correlation of protein pairs in the nucleoplasm by DC-FCCS**. Original Data from which “means” and “SD” values were calculated and presented in Table 1.

| EGFP-(l)-CENP-M  mCherry-(s)-CENP-K | EGFP-(l)-CENP-M  mCherry-(s)-CENP-I | EGFP-(s)-CENP-I  mCherry-(s)-CENP-M | CENP-I-(s)-EGFP  CENP-M-(s)-mCherry |
| --- | --- | --- | --- |
| 51.0000  34.0000  39.0000  29.0000  31.0000  49.0000  50.0000  47.0000  45.0000  46.0000  29.0000  31.0000  31.0000  32.0000  42.0000  27.0000  28.0000  27.0000  26.0000 | 31.0000  20.0000  29.0000  37.0000  25.0000  22.0000  14.0000  25.0000  20.0000  16.0000  19.0000  34.0000  30.0000  16.0000  20.0000  43.0000  31.0000  42.0000  36.0000  18.0000  26.0000  17.0000  14.0000  15.0000  14.0000  35.0000  27.0000  16.0000  16.0000  18.0000  19.0000  24.0000 | 0.0000  0.0000  0.0000  0.0000  0.0000  0.0000  0.0000  0.0000  1.0000  0.0000  1.0000  0.0000 | 0.0000  0.0000  1.0000  0.0000  0.0000  0.0000  0.0000  0.0000  0.0000  0.0000 |

| EGFP-(s)-CENP-H  CENP-I-(s)-mCherry | EGFP-(s)-CENP-H  mCherry-(s)-CENP-K | EGFP-(s)-CENP-H  CENP-C-(s)-mCherry | EGFP-(s)-CENP-H  mCherry-(s)-CENP-C |
| --- | --- | --- | --- |
| 18.0000  17.0000  22.0000  21.0000  20.0000  16.0000  19.0000  28.0000  22.0000  16.0000  20.0000  20.0000  16.0000  16.0000  14.0000  21.0000  24.0000  28.0000  35.0000  36.0000  20.0000  23.0000  19.0000  14.0000  23.0000  32.0000 | 24.0000  33.0000  27.0000  36.0000  37.0000  31.0000  50.0000  25.0000  34.0000  30.0000  39.0000  31.0000  25.0000  29.0000  50.0000  29.0000  27.0000  27.0000  23.0000  35.0000  26.0000  23.0000  22.0000 | 19.0000  9.0000  32.0000  25.0000  34.0000  12.0000  9.0000  9.0000  8.0000  18.0000  25.0000 | 26.0000  27.0000  33.0000  15.0000  32.0000  31.0000  27.0000  34.0000  18.0000  25.0000  24.0000  12.0000  25.0000  33.0000  23.0000  31.0000  27.0000  37.0000  34.0000  37.0000 |

| EGFP-(s)-CENP-K  mCherry-(s)-CENP-I | CENP-K-(s)-EGFP  CENP-T-(s)-mCherry |
| --- | --- |
| 35.0000  25.0000  20.0000  16.0000  19.0000  27.0000  35.0000  16.0000  14.0000  12.0000  28.0000  22.0000  19.0000  16.0000  18.0000  13.0000  13.0000  18.0000  16.0000 | 18.0000  20.0000  22.0000  30.0000  50.0000  55.0000  58.0000  44.0000  47.0000  20.0000  24.0000  29.0000  30.0000  32.0000  25.0000  20.0000  22.0000  24.0000  22.0000  26.0000 |

| EGFP-(l)-CENP-L  mCherry-(s)-CENP-N | EGFP-(l)-CENP-L  mCherry-(s)-CENP-K | EGFP-(s)-CENP-N  mCherry-(s)-CENP-K | CENP-K-(s)-EGFP  mCherry-(s)-CENP-N |
| --- | --- | --- | --- |
| 35.0000  47.0000  28.0000  20.0000  24.0000  25.0000  15.0000  17.0000  15.0000  21.0000  19.0000  16.0000  27.0000  14.0000  19.0000  18.0000 | 16.0000  12.0000  14.0000  19.0000  10.0000  15.0000  36.0000  25.0000  36.0000  19.0000  16.0000  24.0000  14.0000  12.0000  22.0000  11.0000  23.0000  16.0000  11.0000  14.0000  18.0000  16.0000 | 0.0000  0.0000  0.0000  0.0000 | 17.0000  11.0000  24.0000  10.0000  11.0000  19.0000  25.0000  37.0000  46.0000  37.0000  46.0000  37.0000  41.0000  9.0000  11.0000  17.0000  28.0000  39.0000  36.0000 |

| EGFP-(s)-CENP-T  mCherry-(s)-CENP-S | EGFP-(s)-CENP-T  CENP-S-(s)-mCherry | EGFP-(s)-CENP-T  mCherry-(l)-CENP-S | EGFP-(l)-CENP-T  CENP-S-(l)-mCherry |
| --- | --- | --- | --- |
| 0.0000  0.0000  0.0000  0.0000  0.0000  0.0000  0.0000  0.0000  0.0000  1.0000  0.0000  0.0000  0.0000  0.0000 | 0.0000  0.0000  1.0000  0.0000  0.0000  1.0000  0.0000 | 0.0000  1.0000  0.0000  0.0000  0.0000  0.0000 | 0.0000  0.0000  0.0000  0.0000  1.0000  0.0000 |

| CENP-T-(s)-EGFP  mCherry-(s)-CENP-W | EGFP-(s)-CENP-T  mCherry-(s)-CENP-X | EGFP-(l)-CENP-W  CENP-X-(l)-mCherry | EGFP-(s)-CENP-X  mCheryy-(l)-CENP-S |
| --- | --- | --- | --- |
| 21.0000  22.0000  20.0000  31.0000  21.0000  45.0000  43.0000  38.0000  34.0000  35.0000  21.0000  25.0000  28.0000  26.0000  26.0000  27.0000  26.0000  17.0000 | 0.0000  0.0000  0.0000  1.0000  0.0000  0.0000  0.0000  0.0000  0.0000  1.0000 | 1.0000  0.0000  0.0000  1.0000 | 42.0000  38.0000  36.0000  31.0000  29.0000  27.0000  33.0000  24.0000  20.0000 |

| EGFP-(s)-CENP-M  CENP-T^ΔC^-(s)-mCherry | EGFP-(s)-CENP-M  CENP-T-(s)-mCherry | EGFP-(s)-Dsn1  mCherry-(s)-Nnf1 | EGFP-(s)-Nsl1  mCherry-(s)-Nnf1 |
| --- | --- | --- | --- |
| 1.0000  0.0000  0.0000  0.0000  0.0000  0.0000  0.0000  0.0000  0.0000  0.0000  1.0000  0.0000  0.0000  0.0000  0.0000 | 10.0000  13.0000  15.0000  14.0000 | 21.0000  20.0000  22.0000  21.0000  18.0000  17.0000  23.0000  20.0000  24.0000  25.0000  24.0000  24.0000  26.0000  19.0000  27.0000  24.0000  19.0000  27.0000 | 10.0000  22.0000  13.0000  11.0000  18.0000  26.0000  24.0000  20.0000  27.0000  27.0000  17.0000  17.0000  18.0000  16.0000  11.0000 |

| EGFP-(s)-Dsn1  mCherry-(s)-Nsl1 | hMis12-(s)-EGFP  Nnf1-(s)-mCherry | Nsl1-(s)-EGFP  hMis12-(s)-mCherry | EGFP-(s)-Dsn1  hMis12-(s)-mCherry |
| --- | --- | --- | --- |
| 25.0000  24.0000  30.0000  20.0000  23.0000  25.0000  34.0000  16.0000  14.0000  25.0000  21.0000  29.0000  26.0000 | 23.0000  18.0000  18.0000  39.0000  38.0000  35.0000  39.0000  33.0000  34.0000  38.0000  30.0000  25.0000  29.0000  30.0000  21.0000  26.0000  33.0000  33.0000  37.0000  49.0000  28.0000  23.0000 | 27.0000  41.0000  46.0000  48.0000  46.0000  42.0000  31.0000  41.0000  41.0000  27.0000  42.0000  25.0000  24.0000  48.0000  48.0000  42.0000  36.0000  15.0000  15.0000 | 22.0000  30.0000  32.0000  32.0000  26.0000  33.0000  20.0000  42.0000  38.0000  33.0000  39.0000  43.0000  39.0000  40.0000  24.0000  33.0000  21.0000  37.0000 |

| hMis12-(s)-EGFP  mCherry-(s)-CENP-K | hMis12-(s)-EGFP  mCherry-(s)-CENP-T | hMis12-(s)-EGFP  CENP-T-(s)-mCherry |
| --- | --- | --- |
| 19.0000  23.0000  15.0000  15.0000  20.0000  16.0000  21.0000  13.0000  20.0000  21.0000  23.0000  26.0000  10.0000  17.0000  15.0000  12.0000  21.0000  17.0000  24.0000  20.0000  15.0000  10.0000 | 20.0000  24.0000  17.0000  14.0000  12.0000  15.0000  19.0000  22.0000  14.0000  13.0000  22.0000  21.0000  21.0000  21.0000  12.0000  14.0000  20.0000 | 25.0000  19.0000  22.0000  14.0000  17.0000  16.0000  14.0000  22.0000 |
