# Supplementary material for: CENP-C/H/I/K/M/T/W/N/L and hMis12 but not CENP-S/X participate in complex formation in the nucleoplasm of living human interphase cells outside centromeres
Source: PLoS One. 2018 Mar 6;13(3):e0192572. doi: 10.1371/journal.pone.0192572 (PMC5839545; doi:10.1371/journal.pone.0192572)
Supplement: S2 Table — Original Data from which “means” and “SD” values were calculated and presented in Table 2. (DOCX) [file pone.0192572.s002.docx]

**S2 Table. Cross-correlation of protein pairs in the nucleoplasm by SW-FCCS.** Original

data from which “means” and “SD” were calculated and presented in table 2.

| EGFP-(s)-Spc24/mCherry-(s)-CENP-T | | EGFP-(s)-CENP-S/mCherry-(s)-CENP-W | | EGFP-(s)-Spc24/mCherry-(s)-CENPT^ΔN^ | |
| --- | --- | --- | --- | --- | --- |
| τ_D_ ± SD (s) (G) | τ_D_ ± SD (s) (R) | τ_D_ ± SD (s) (G) | τ_D_ ± SD (s) (R) | τ_D_ ± SD (s) (G) | τ_D_ ± SD (s) (R) |
| \| \| 0.01026 \| \| --- \| \| 0.01998 \| \| 0.007249 \| \| 0.005844 \| \| 0.0235 \| \| 0.007582 \| \| 0.006148 \| \| 0.007368 \| \| 0.0184 \| \| 0.009822 \| \| 0.01034 \| \| 0.003036 \| \| 0.004151 \| \| 0.002492 \| \| 0.01438 \| \| 0.003668 \| \| 0.003472 \| \| 0.004688 \| \| 0.02206 \| \| 0.008709 \| \| 0.005374 \| \| 0.01008 \| \| 0.006031 \| \| 0.00341 \| \| 0.0126 \| \| 0.005006 \| \| 0.004162 \| \| 0.006566 \| \| 0.008342 \| \| 0.01316 \| \| 0.1406 \| \| 0.004374 \| \| 0.005213 \| \| 0.01448 \| \| 0.01096 \| \| 0.00385 \| \| 0.005197 \| \| 0.003568 \| \| 0.006835 \| \| 0.005423 \| \| 0.003438 \| \| 0.003979 \| \| 0.005344 \| \| 0.002565 \| \| 0.002319 \| \| 0.006099 \| \| 0.01012 \| \| 0.004764 \| \| 0.04057 \| \| 0.005477 \| \| 0.02678 \| \| 0.003644 \| \| 0.00264 \| \| 0.004139 \| \| 0.01789 \| \| 0.008162 \| \| 0.01653 \| \| 0.01356 \| \| 0.01474 \| \| 0.003018 \| \| 0.005811 \| \| 0.01938 \| \| 0.007928 \| \| 0.007152 \| \| 0.009266 \| \| 0.006526 \| \| 0.008634 \| \| 0.01985 \| \| 0.01244 \| \| 0.0164 \| \| 0.005511 \| \| 0.004817 \| \| 0.003621 \| \| 0.006579 \| \| 0.0147 \| \| 0.008564 \| \| 0.007778 \| \| 0.05405 \| \| 0.06775 \| \| 0.004288 \| \| 0.006994 \| \| 0.02213 \| \| 0.008601 \| \| 0.01022 \| \| 0.002671 \| \| 0.009852 \| \| \| --- \| --- \| --- \| --- \| --- \| --- \| --- \| --- \| --- \| --- \| --- \| --- \| --- \| --- \| --- \| --- \| --- \| --- \| --- \| --- \| --- \| --- \| --- \| --- \| --- \| --- \| --- \| --- \| --- \| --- \| --- \| --- \| --- \| --- \| --- \| --- \| --- \| --- \| --- \| --- \| --- \| --- \| --- \| --- \| --- \| --- \| --- \| --- \| --- \| --- \| --- \| --- \| --- \| --- \| --- \| --- \| --- \| --- \| --- \| --- \| --- \| --- \| --- \| --- \| --- \| --- \| --- \| --- \| --- \| --- \| --- \| --- \| --- \| --- \| --- \| --- \| --- \| --- \| --- \| --- \| --- \| --- \| --- \| --- \| --- \| --- \| --- \| | \| 0.003129 \| \| --- \| \| 0.00329 \| \| 0.002382 \| \| 0.003497 \| \| 0.004894 \| \| 0.003726 \| \| 0.004905 \| \| 0.002665 \| \| 0.003123 \| \| 0.004285 \| \| 0.004358 \| \| 0.003956 \| \| 0.01299 \| \| 0.01552 \| \| 0.01267 \| \| 0.006147 \| \| 0.00804 \| \| 0.003086 \| \| 0.01028 \| \| 0.01028 \| \| 0.006119 \| \| 0.007727 \| \| 0.01109 \| \| 0.01004 \| \| 0.00557 \| \| 0.007889 \| \| 0.009898 \| \| 0.005555 \| \| 0.01686 \| \| 0.01773 \| \| 0.002196 \| \| 0.002346 \| \| 0.00662 \| \| 0.005813 \| \| 0.006343 \| \| 0.003564 \| \| 0.003656 \| \| 0.00257 \| \| 0.00419 \| \| 0.004899 \| \| 0.00399 \| \| 0.003243 \| \| 0.002453 \| \| 0.002584 \| \| 0.004646 \| \| 0.01065 \| \| 0.01175 \| \| 0.01617 \| \| 0.01236 \| \| 0.02075 \| \| 0.01212 \| \| 0.006618 \| \| 0.008999 \| \| 0.01184 \| \| 0.003298 \| \| 0.008705 \| \| 0.006878 \| \| 0.007209 \| \| 0.005805 \| \| 0.007472 \| \| 0.007428 \| \| 0.008291 \| \| 0.007587 \| \| 0.01881 \| \| 0.00984 \| \| 0.008499 \| \| 0.009424 \| \| 0.009051 \| \| 0.01109 \| \| 0.02849 \| \| 0.007903 \| \| 0.007893 \| \| 0.01717 \| \| 0.009445 \| \| 0.008133 \| \| 0.007066 \| \| 0.01063 \| \| 0.008256 \| \| 0.01128 \| \| 0.01212 \| \| 0.009883 \| \| 0.004514 \| \| 0.01209 \| \| 0.007545 \| \| 0.007155 \| \| 0.006155 \| | \| 0.00511 \| \| --- \| \| 0.008874 \| \| 0.004452 \| \| 0.004689 \| \| 0.002517 \| \| 0.01311 \| \| 0.006158 \| \| 0.007248 \| \| 0.0028 \| \| 0.003909 \| \| 0.005127 \| \| 0.003458 \| \| 0.01251 \| \| 0.00192 \| \| 0.001948 \| \| 0.002131 \| \| 0.002984 \| \| 0.00161 \| \| 0.001538 \| \| 0.006435 \| \| 0.002319 \| \| 0.000625 \| \| 0.000872 \| \| 0.001304 \| \| 0.002638 \| \| 0.001855 \| \| 0.002277 \| \| 0.001453 \| \| 0.004067 \| \| 0.001458 \| \| 0.003232 \| \| 0.001315 \| \| 0.002652 \| \| 0.001205 \| \| 0.001905 \| \| 0.001649 \| \| 0.001176 \| \| 0.002103 \| \| 0.003607 \| \| 0.001335 \| \| 0.000772 \| \| 0.002009 \| \| 0.001843 \| | \| 0.0026 \| \| --- \| \| 0.001461 \| \| 0.00134 \| \| 0.001635 \| \| 0.001637 \| \| 0.002615 \| \| 0.002754 \| \| 0.00416 \| \| 0.00135 \| \| 0.001209 \| \| 0.000811 \| \| 0.002452 \| \| 0.002418 \| \| 0.001434 \| \| 0.001078 \| \| 0.001049 \| \| 0.000768 \| \| 0.003117 \| \| 0.004913 \| \| 0.001314 \| \| 0.00094 \| \| 0.000356 \| \| 0.001499 \| \| 0.000672 \| \| 0.000406 \| \| 0.000439 \| \| 0.000621 \| \| 0.000753 \| \| 0.000907 \| \| 0.001313 \| \| 0.000764 \| \| 0.000572 \| \| 0.001322 \| \| 0.000417 \| \| 0.00081 \| \| 0.000373 \| \| 0.000631 \| \| 0.001074 \| \| 0.002491 \| \| 0.000761 \| \| 0.000669 \| \| 0.001567 \| \| 0.00094 \| | \| 0.01793 \| \| --- \| \| 0.0106 \| \| 0.01814 \| \| 0.02492 \| \| 0.008567 \| \| 0.0297 \| \| 0.005137 \| \| 0.006334 \| \| 0.01555 \| \| 0.01161 \| \| 0.002187 \| \| 0.008447 \| \| 0.008946 \| \| 0.006252 \| \| 0.007653 \| \| 0.002854 \| \| 0.002628 \| \| 0.005336 \| \| 0.01331 \| \| 0.006688 \| \| 0.003977 \| \| 0.005305 \| \| 0.02535 \| \| 0.005991 \| \| 0.01205 \| | \| 0.009898 \| \| --- \| \| 0.008354 \| \| 0.007678 \| \| 0.004826 \| \| 0.007641 \| \| 0.009609 \| \| 0.005078 \| \| 0.004579 \| \| 0.00695 \| \| 0.003192 \| \| 0.003971 \| \| 0.006433 \| \| 0.003935 \| \| 0.00352 \| \| 0.004032 \| \| 0.002701 \| \| 0.005549 \| \| 0.00341 \| \| 0.0019 \| \| 0.002106 \| \| 0.004042 \| \| 0.002618 \| \| 0.01347 \| \| 0.004001 \| \| 0.004621 \| |

| mCherry-EGFP | | mCherry-(s)-CENP-T/EGFP-(s)-CENP-X | | EGFP/mCherry | |
| --- | --- | --- | --- | --- | --- |
| τ_D_ ± SD (s) (G) | τ_D_ ± SD (s) (R) | τ_D_ ± SD (s) (G) | τ_D_ ± SD (s) (R) | τ_D_ ± SD (s) (G) | τ_D_ ± SD (s) (R) |
| \| 0.004694 \| \| --- \| \| 0.001815 \| \| 0.001312 \| \| 0.001658 \| \| 0.001445 \| \| 0.003213 \| \| 0.001575 \| \| 0.001312 \| \| 0.00135 \| \| 0.001108 \| \| 0.001298 \| \| 0.001463 \| \| 0.001388 \| \| 0.0008 73 \| \| 0.001395 \| \| 0.0007633 \| \| 0.003197 \| \| 0.007839 \| \| 0.002153 \| \| 0.00298 \| \| 0.001876 \| \| 0.002664 \| \| 0.002711 \| \| 0.001949 \| \| 0.002608 \| \| 0.001802 \| \| 0.0007772 \| \| 0.0007915 \| \| 0.0008792 \| \| 0.0009643 \| \| 0.001051 \| \| 0.001823 \| \| 0.001467 \| \| 0.001373 \| \| 0.001479 \| \| 0.001456 \| \| 0.001748 \| \| 0.002182 \| \| 0.001995 \| \| 0.001564 \| \| 0.001492 \| \| 0.002921 \| \| 0.002021 \| \| 0.001558 \| \| 0.002508 \| \| 0.002253 \| \| 0.002306 \| | \| 0.002161 \| \| --- \| \| 0.002419 \| \| 0.001141 \| \| 0.001683 \| \| 0.00181 \| \| 0.001202 \| \| 0.000808 \| \| 0.001018 \| \| 0.0009742 \| \| 0.0008985 \| \| 0.0007557 \| \| 0.001232 \| \| 0.001155 \| \| 0.0005331 \| \| 0.0007754 \| \| 0.0007193 \| \| 0.0009966 \| \| 0.001086 \| \| 0.001162 \| \| 0.001462 \| \| 0.001114 \| \| 0.00142 \| \| 0.001294 \| \| 0.001284 \| \| 0.001919 \| \| 0.001591 \| \| 0.0007022 \| \| 0.0006385 \| \| 0.0009645 \| \| 0.0006452 \| \| 0.001417 \| \| 0.001098 \| \| 0.001149 \| \| 0.001152 \| \| 0.001561 \| \| 0.001314 \| \| 0.00165 \| \| 0.001507 \| \| 0.001401 \| \| 0.001133 \| \| 0.00101 \| \| 0.002108 \| \| 0.002002 \| \| 0.00184 \| \| 0.001285 \| \| 0.00122 \| \| 0.001172 \| | \| 0.004503 \| \| --- \| \| 0.002624 \| \| 0.008986 \| \| 0.001634 \| \| 0.001809 \| \| 0.000791 \| \| 0.000847 \| \| 0.002454 \| \| 0.007777 \| \| 0.006007 \| \| 0.002747 \| \| 0.002244 \| \| 0.00086 \| \| 0.00147 \| \| 0.001361 \| \| 0.001451 \| \| 0.002028 \| \| 0.001352 \| \| 0.001053 \| \| 0.000852 \| \| 0.001387 \| \| 0.000918 \| \| 0.000835 \| \| 0.002529 \| \| 0.009625 \| \| 0.001121 \| \| 0.002275 \| \| 0.000952 \| \| 0.0022 \| \| 0.001594 \| \| 0.001629 \| \| 0.001171 \| \| 0.002665 \| \| 0.003718 \| \| 0.002307 \| \| 0.001844 \| \| 0.004512 \| \| 0.002699 \| \| 0.003619 \| \| 0.001908 \| \| 0.000953 \| \| 0.002018 \| \| 0.001877 \| \| 0.001758 \| \| 0.003656 \| \| 0.001212 \| \| 0.002406 \| \| 0.002433 \| \| 0.000823 \| \| 0.001218 \| \| 0.00085 \| \| 0.003205 \| \| 0.003905 \| \| 0.001719 \| | \| 0.004943 \| \| --- \| \| 0.002773 \| \| 0.002239 \| \| 0.002242 \| \| 0.002097 \| \| 0.002019 \| \| 0.001657 \| \| 0.002163 \| \| 0.002388 \| \| 0.001466 \| \| 0.00396 \| \| 0.0024 \| \| 0.003183 \| \| 0.00334 \| \| 0.002753 \| \| 0.002993 \| \| 0.006884 \| \| 0.00224 \| \| 0.003449 \| \| 0.001948 \| \| 0.003435 \| \| 0.002346 \| \| 0.001684 \| \| 0.003658 \| \| 0.003093 \| \| 0.002718 \| \| 0.003717 \| \| 0.002974 \| \| 0.002198 \| \| 0.002466 \| \| 0.002118 \| \| 0.003487 \| \| 0.002144 \| \| 0.001712 \| \| 0.002817 \| \| 0.004226 \| \| 0.006842 \| \| 0.003473 \| \| 0.004537 \| \| 0.004909 \| \| 0.003724 \| \| 0.003356 \| \| 0.003273 \| \| 0.001776 \| \| 0.005455 \| \| 0.002904 \| \| 0.001825 \| \| 0.001993 \| \| 0.002059 \| \| 0.001715 \| \| 0.001261 \| \| 0.001005 \| \| 0.002602 \| \| 0.002508 \| | \| 0.004354 \| \| --- \| \| 0.002746 \| \| 0.005355 \| \| 0.002137 \| \| 0.000753 \| \| 0.0008752 \| \| 0.001727 \| \| 0.00172 \| \| 0.0005738 \| \| 0.0007002 \| \| 0.001307 \| \| 0.001577 \| \| 0.001021 \| \| 0.002525 \| \| 0.001493 \| \| 0.0003017 \| \| 0.0007728 \| \| 0.001507 \| \| 0.0009536 \| | \| 0.004777 \| \| --- \| \| 0.005479 \| \| 0.004248 \| \| 0.0008195 \| \| 0.0008299 \| \| 0.0007263 \| \| 0.00112 \| \| 0.001154 \| \| 0.0003446 \| \| 0.0003207 \| \| 0.001093 \| \| 0.00112 \| \| 0.0008458 \| \| 0.001052 \| \| 0.0007081 \| \| 0.000636 \| \| 0.0006872 \| \| 0.0007773 \| \| 0.001572 \| |

| EGFP-(s)-CENP-T^ΔN^/mCherry-(s)-CENP-W | | EGFP-(s)-CENP-W/mCherry-(s)-CENP-T | | EGFP-(s)-CENP-S/mCherry-(s)-CENP-X | |
| --- | --- | --- | --- | --- | --- |
| τ_D_ ± SD (s) (G) | τ_D_ ± SD (s) (R) | τ_D_ ± SD (s) (G) | τ_D_ ± SD (s) (R) | τ_D_ ± SD (s) (G) | τ_D_ ± SD (s) (R) |
| \| 0.002848 \| \| --- \| \| 0.001942 \| \| 0.002616 \| \| 0.001651 \| \| 0.00249 \| \| 0.004304 \| \| 0.004018 \| \| 0.002208 \| \| 0.002063 \| \| 0.003094 \| \| 0.001659 \| \| 0.003524 \| \| 0.004411 \| \| 0.005818 \| \| 0.003097 \| \| 0.005188 \| \| 0.001895 \| \| 0.00345 \| \| 0.00213 \| \| 0.001493 \| \| 0.002079 \| \| 0.000847 \| \| 0.001166 \| \| 0.000859 \| \| 0.001298 \| \| 0.001727 \| \| 0.003225 \| \| 0.001361 \| \| 0.002163 \| \| 0.002065 \| \| 0.001464 \| \| 0.002665 \| \| 0.003198 \| \| 0.003701 \| \| 0.003365 \| \| 0.003556 \| \| 0.006627 \| \| 0.002038 \| \| 0.003203 \| \| 0.002319 \| \| 0.006181 \| \| 0.003172 \| \| 0.001812 \| \| 0.004252 \| \| 0.003379 \| \| 0.005487 \| \| 0.004691 \| \| 0.006097 \| \| 0.003075 \| \| 0.004296 \| \| 0.003285 \| \| 0.003534 \| \| 0.005244 \| \| 0.003682 \| \| 0.000961 \| \| 0.000991 \| \| 0.002975 \| \| 0.005911 \| \| 0.00299 \| \| 0.01269 \| \| 0.006117 \| \| 0.003068 \| \| 0.002471 \| \| 0.001612 \| \| 0.002653 \| \| 0.001411 \| \| 0.003831 \| \| 0.001386 \| \| 0.000934 \| \| 0.001072 \| \| 0.002364 \| \| 0.002518 \| \| 0.004369 \| \| 0.003321 \| \| 0.004358 \| \| 0.003279 \| \| 0.002643 \| \| 0.002519 \| \| 0.002347 \| \| 0.003136 \| \| 0.004071 \| \| 0.00263 \| \| 0.004104 \| \| 0.002775 \| \| 0.003023 \| \| 0.007925 \| \| 0.004234 \| \| 0.006452 \| \| 0.005193 \| \| 0.003515 \| \| 0.002138 \| \| 0.001365 \| \| 0.001896 \| \| 0.002031 \| \| 0.001856 \| \| 0.008809 \| \| 0.002304 \| \| 0.001858 \| \| 0.002521 \| \| 0.002481 \| \| 0.002218 \| \| 0.005205 \| \| 0.008197 \| \| 0.002833 \| \| 0.00449 \| \| 0.00858 \| \| 0.011 \| \| 0.008611 \| \| 0.01009 \| \| 0.01184 \| \| 0.007367 \| \| 0.005945 \| \| 0.006188 \| \| 0.009547 \| \| 0.001277 \| \| 0.001848 \| \| 0.002761 \| \| 0.0026 \| \| 0.004927 \| \| 0.006398 \| \| 0.001774 \| \| 0.00193 \| \| 0.001789 \| | \| 0.001192 \| \| --- \| \| 0.000743 \| \| 0.002199 \| \| 0.001045 \| \| 0.002061 \| \| 0.001676 \| \| 0.000753 \| \| 0.002004 \| \| 0.00164 \| \| 0.000704 \| \| 0.001996 \| \| 0.002366 \| \| 0.003708 \| \| 0.00421 \| \| 0.001748 \| \| 0.004197 \| \| 0.002339 \| \| 0.001391 \| \| 0.002048 \| \| 0.002213 \| \| 0.002252 \| \| 0.002366 \| \| 0.00109 \| \| 0.001001 \| \| 0.001106 \| \| 0.002175 \| \| 0.002666 \| \| 0.002357 \| \| 0.001379 \| \| 0.001211 \| \| 0.001213 \| \| 0.00144 \| \| 0.00215 \| \| 0.002219 \| \| 0.002425 \| \| 0.003075 \| \| 0.002879 \| \| 0.001423 \| \| 0.001553 \| \| 0.001278 \| \| 0.002198 \| \| 0.003217 \| \| 0.002879 \| \| 0.002376 \| \| 0.002413 \| \| 0.00355 \| \| 0.001748 \| \| 0.001914 \| \| 0.003671 \| \| 0.003267 \| \| 0.003119 \| \| 0.003801 \| \| 0.002815 \| \| 0.002454 \| \| 0.001742 \| \| 0.002031 \| \| 0.005043 \| \| 0.004594 \| \| 0.005113 \| \| 0.006087 \| \| 0.002701 \| \| 0.003659 \| \| 0.00774 \| \| 0.001951 \| \| 0.002929 \| \| 0.001575 \| \| 0.002189 \| \| 0.001993 \| \| 0.001499 \| \| 0.001409 \| \| 0.002139 \| \| 0.002344 \| \| 0.003518 \| \| 0.002983 \| \| 0.002968 \| \| 0.0027 \| \| 0.002581 \| \| 0.002051 \| \| 0.002297 \| \| 0.001888 \| \| 0.003219 \| \| 0.002867 \| \| 0.002601 \| \| 0.002337 \| \| 0.002485 \| \| 0.003588 \| \| 0.003557 \| \| 0.003963 \| \| 0.00653 \| \| 0.002472 \| \| 0.001629 \| \| 0.002883 \| \| 0.003531 \| \| 0.001987 \| \| 0.002427 \| \| 0.008884 \| \| 0.002877 \| \| 0.001157 \| \| 0.000758 \| \| 0.002638 \| \| 0.000945 \| \| 0.002526 \| \| 0.01103 \| \| 0.001688 \| \| 0.000858 \| \| 0.006333 \| \| 0.007113 \| \| 0.003413 \| \| 0.007038 \| \| 0.004149 \| \| 0.005393 \| \| 0.003962 \| \| 0.004592 \| \| 0.005042 \| \| 0.002192 \| \| 0.001242 \| \| 0.002004 \| \| 0.002439 \| \| 0.005149 \| \| 0.004319 \| \| 0.001199 \| \| 0.000873 \| \| 0.001329 \| | \| \| 0.009883 \| \| --- \| \| 0.01055 \| \| 0.02296 \| \| 0.006415 \| \| 0.02052 \| \| 0.01001 \| \| 0.01688 \| \| 0.01187 \| \| 0.007884 \| \| 0.01077 \| \| 0.0211 \| \| 0.02878 \| \| 0.01662 \| \| 0.01435 \| \| 0.008156 \| \| 0.004849 \| \| 0.003095 \| \| 0.004168 \| \| 0.005808 \| \| 0.008812 \| \| 0.003805 \| \| 0.004339 \| \| 0.024 \| \| 0.03614 \| \| 0.004674 \| \| 0.00138 \| \| 0.005619 \| \| 0.001126 \| \| 0.003594 \| \| 0.002537 \| \| 0.004287 \| \| 0.002035 \| \| 0.001485 \| \| 0.001748 \| \| 0.007188 \| \| 0.003895 \| \| 0.003616 \| \| 0.003379 \| \| 0.002046 \| \| 0.002446 \| \| 0.004047 \| \| 0.006545 \| \| 0.007127 \| \| 0.009363 \| \| 0.007994 \| \| 0.003516 \| \| 0.004156 \| \| 0.007923 \| \| 0.003626 \| \| 0.003999 \| \| 0.004663 \| \| 0.004796 \| \| 0.007736 \| \| 0.005022 \| \| 0.003006 \| \| 0.003108 \| \| 0.005565 \| \| 0.00631 \| \| 0.003114 \| \| 0.007574 \| \| 0.008054 \| \| 0.006646 \| \| 0.006766 \| \| 0.01928 \| \| 0.004764 \| \| 0.008234 \| \| 0.01202 \| \| 0.01699 \| \| 0.01542 \| \| 0.008861 \| \| 0.001835 \| \| 0.001741 \| \| 0.005502 \| \| 0.01701 \| \| 0.01231 \| \| 0.002287 \| \| 0.01538 \| \| 0.002103 \| \| 0.007571 \| \| 0.003836 \| \| 0.01598 \| \| 0.02323 \| \| 0.006037 \| \| 0.003629 \| \| 0.006646 \| \| 0.002854 \| \| 0.002328 \| \| 0.003787 \| \| 0.002991 \| \| 0.002829 \| \| 0.005775 \| \| 0.004681 \| \| 0.003801 \| \| 0.005927 \| \| 0.002654 \| \| 0.002229 \| \| 0.004755 \| \| 0.005238 \| \| 0.002966 \| \| 0.004059 \| \| 0.003379 \| \| 0.003161 \| \| 0.003711 \| \| 0.003561 \| \| 0.005728 \| \| 0.007463 \| \| 0.008178 \| \| 0.001714 \| \| 0.00268 \| \| 0.001959 \| \| 0.001763 \| \| 0.004505 \| \| 0.002899 \| \| 0.00252 \| \| 0.005047 \| \| 0.002795 \| \| 0.002964 \| \| 0.003245 \| \| 0.005036 \| \| 0.005043 \| \| 0.005679 \| \| 0.002577 \| \| 0.006139 \| \| 0.003961 \| \| 0.006936 \| \| 0.005449 \| \| 0.003618 \| \| 0.003643 \| \| 0.004681 \| \| 0.004415 \| \| 0.006095 \| \| 0.003774 \| \| 0.004775 \| \| 0.003173 \| \| 0.002773 \| \| 0.002801 \| \| 0.003043 \| \| 0.003154 \| \| 0.002701 \| \| 0.003522 \| \| 0.005594 \| \| 0.004261 \| \| 0.008589 \| \| 0.005639 \| \| 0.005776 \| \| 0.004817 \| \| 0.01231 \| \| 0.01359 \| \| 0.01118 \| \| 0.01555 \| \| 0.01545 \| \| 0.0513 \| \| \| --- \| --- \| --- \| --- \| --- \| --- \| --- \| --- \| --- \| --- \| --- \| --- \| --- \| --- \| --- \| --- \| --- \| --- \| --- \| --- \| --- \| --- \| --- \| --- \| --- \| --- \| --- \| --- \| --- \| --- \| --- \| --- \| --- \| --- \| --- \| --- \| --- \| --- \| --- \| --- \| --- \| --- \| --- \| --- \| --- \| --- \| --- \| --- \| --- \| --- \| --- \| --- \| --- \| --- \| --- \| --- \| --- \| --- \| --- \| --- \| --- \| --- \| --- \| --- \| --- \| --- \| --- \| --- \| --- \| --- \| --- \| --- \| --- \| --- \| --- \| --- \| --- \| --- \| --- \| --- \| --- \| --- \| --- \| --- \| --- \| --- \| --- \| --- \| --- \| --- \| --- \| --- \| --- \| --- \| --- \| --- \| --- \| --- \| --- \| --- \| --- \| --- \| --- \| --- \| --- \| --- \| --- \| --- \| --- \| --- \| --- \| --- \| --- \| --- \| --- \| --- \| --- \| --- \| --- \| --- \| --- \| --- \| --- \| --- \| --- \| --- \| --- \| --- \| --- \| --- \| --- \| --- \| --- \| --- \| --- \| --- \| --- \| --- \| --- \| --- \| --- \| --- \| --- \| --- \| --- \| --- \| --- \| --- \| --- \| --- \| --- \| --- \| --- \| \|  \| | \| 0.006763 \| \| --- \| \| 0.001283 \| \| 0.001701 \| \| 0.001954 \| \| 0.002861 \| \| 0.00298 \| \| 0.00473 \| \| 0.008415 \| \| 0.003102 \| \| 0.005376 \| \| 0.005365 \| \| 0.004119 \| \| 0.004235 \| \| 0.003801 \| \| 0.002555 \| \| 0.002181 \| \| 0.003678 \| \| 0.00314 \| \| 0.003682 \| \| 0.0061 \| \| 0.006355 \| \| 0.002876 \| \| 0.002261 \| \| 0.00338 \| \| 0.003816 \| \| 0.002384 \| \| 0.002592 \| \| 0.006566 \| \| 0.003328 \| \| 0.00544 \| \| 0.004062 \| \| 0.002665 \| \| 0.003967 \| \| 0.002636 \| \| 0.00408 \| \| 0.003773 \| \| 0.003744 \| \| 0.002968 \| \| 0.003404 \| \| 0.004054 \| \| 0.004727 \| \| 0.005207 \| \| 0.006877 \| \| 0.0045 \| \| 0.003537 \| \| 0.00315 \| \| 0.007127 \| \| 0.008041 \| \| 0.006514 \| \| 0.02548 \| \| 0.01067 \| \| 0.01121 \| \| 0.007426 \| \| 0.01116 \| \| 0.007825 \| \| 0.01383 \| \| 0.00763 \| \| 0.005215 \| \| 0.004834 \| \| 0.007771 \| \| 0.006226 \| \| 0.006362 \| \| 0.02011 \| \| 0.02297 \| \| 0.00326 \| \| 0.004518 \| \| 0.004454 \| \| 0.006638 \| \| 0.005274 \| \| 0.005779 \| \| 0.001151 \| \| 0.001552 \| \| 0.002475 \| \| 0.00311 \| \| 0.003604 \| \| 0.001325 \| \| 0.002582 \| \| 0.001367 \| \| 0.00238 \| \| 0.002435 \| \| 0.007271 \| \| 0.009405 \| \| 0.001967 \| \| 0.001787 \| \| 0.002558 \| \| 0.003574 \| \| 0.002958 \| \| 0.003277 \| \| 0.002581 \| \| 0.002724 \| \| 0.005598 \| \| 0.00421 \| \| 0.003217 \| \| 0.004878 \| \| 0.00325 \| \| 0.003757 \| \| 0.004002 \| \| 0.002726 \| \| 0.004963 \| \| 0.00299 \| \| 0.004819 \| \| 0.003369 \| \| 0.002245 \| \| 0.004592 \| \| 0.00436 \| \| 0.009068 \| \| 0.008647 \| \| 0.001964 \| \| 0.003067 \| \| 0.002388 \| \| 0.001565 \| \| 0.004424 \| \| 0.003855 \| \| 0.003848 \| \| 0.003343 \| \| 0.00594 \| \| 0.003713 \| \| 0.004678 \| \| 0.001586 \| \| 0.008153 \| \| 0.003544 \| \| 0.001492 \| \| 0.003938 \| \| 0.003916 \| \| 0.006274 \| \| 0.004653 \| \| 0.003956 \| \| 0.004774 \| \| 0.005633 \| \| 0.003996 \| \| 0.00508 \| \| 0.006593 \| \| 0.005864 \| \| 0.009759 \| \| 0.004728 \| \| 0.009957 \| \| 0.003685 \| \| 0.003852 \| \| 0.004173 \| \| 0.004207 \| \| 0.006105 \| \| 0.004089 \| \| 0.008529 \| \| 0.006414 \| \| 0.006008 \| \| 0.004931 \| \| 0.009085 \| \| 0.009263 \| \| 0.007853 \| \| 0.01496 \| \| 0.01746 \| \| 0.01374 \| | \| 0.002675 \| \| --- \| \| 0.003112 \| \| 0.002705 \| \| 0.003825 \| \| 0.003841 \| \| 0.004751 \| \| 0.00266 \| \| 0.004113 \| \| 0.003661 \| \| 0.008508 \| \| 0.009714 \| \| 0.007025 \| \| 0.001518 \| \| 0.002051 \| \| 0.001186 \| \| 0.006941 \| \| 0.001072 \| \| 0.000879 \| \| 0.000813 \| \| 0.002767 \| \| 0.002949 \| \| 0.002172 \| \| 0.001302 \| \| 0.008228 \| \| 0.007285 \| \| 0.004444 \| \| 0.002841 \| \| 0.005478 \| \| 0.003849 \| \| 0.001908 \| \| 0.002313 \| \| 0.005643 \| \| 0.003467 \| \| 0.004989 \| \| 0.002053 \| \| 0.002957 \| \| 0.002661 \| \| 0.001279 \| \| 0.001862 \| \| 0.001375 \| \| 0.001464 \| \| 0.001253 \| \| 0.001085 \| \| 0.001104 \| \| 0.004499 \| \| 0.001364 \| \| 0.00112 \| \| 0.001147 \| \| 0.001795 \| \| 0.001533 \| \| 0.001387 \| \| 0.002739 \| \| 0.002451 \| \| 0.002185 \| \| 0.002046 \| \| 0.001441 \| \| 0.001626 \| \| 0.002875 \| \| 0.001473 \| \| 0.003175 \| \| 0.001071 \| \| 0.001465 \| \| 0.001628 \| \| 0.001055 \| \| 0.001678 \| \| 0.001214 \| | \| 0.001003 \| \| --- \| \| 0.001703 \| \| 0.002272 \| \| 0.001341 \| \| 0.000923 \| \| 0.001586 \| \| 0.00285 \| \| 0.00631 \| \| 0.004098 \| \| 0.004922 \| \| 0.004672 \| \| 0.008619 \| \| 0.002172 \| \| 0.001396 \| \| 0.00104 \| \| 0.002079 \| \| 0.000962 \| \| 0.000821 \| \| 0.000928 \| \| 0.002423 \| \| 0.001781 \| \| 0.002674 \| \| 0.002253 \| \| 0.004342 \| \| 0.004987 \| \| 0.005354 \| \| 0.002452 \| \| 0.004862 \| \| 0.004421 \| \| 0.001843 \| \| 0.004056 \| \| 0.00451 \| \| 0.003636 \| \| 0.002689 \| \| 0.00235 \| \| 0.002562 \| \| 0.002329 \| \| 0.001397 \| \| 0.002236 \| \| 0.002147 \| \| 0.001779 \| \| 0.001705 \| \| 0.001296 \| \| 0.0014 \| \| 0.002304 \| \| 0.001312 \| \| 0.001732 \| \| 0.000592 \| \| 0.001326 \| \| 0.001276 \| \| 0.00096 \| \| 0.001933 \| \| 0.001558 \| \| 0.002366 \| \| 0.001524 \| \| 0.001416 \| \| 0.001446 \| \| 0.002966 \| \| 0.001743 \| \| 0.002067 \| \| 0.0005 \| \| 0.001538 \| \| 0.000794 \| \| 0.001194 \| \| 0.002292 \| \| 0.000616 \| |

| EGFP-(s)-Spc25/mCherry-(s)-CENP-T^ΔC^ | | EGFP-(s)-CENP-S/mCherry-(s)-CENP-T | | EGFP-(s)-CENP-W/mCherry-(s)-CENP-X | |
| --- | --- | --- | --- | --- | --- |
| τ_D_ ± SD (s) (G) | τ_D_ ± SD (s) (R) | τ_D_ ± SD (s) (G) | τ_D_ ± SD (s) (R) | τ_D_ ± SD (s) (G) | τ_D_ ± SD (s) (R) |
| \| \| 0.02222 \| \| --- \| \| 0.007315 \| \| 0.005113 \| \| 0.003647 \| \| 0.008217 \| \| 0.01028 \| \| 0.007429 \| \| 0.009623 \| \| 0.006562 \| \| 0.004846 \| \| 0.005215 \| \| 0.004132 \| \| 0.005454 \| \| 0.005566 \| \| 0.01626 \| \| 0.01152 \| \| 0.04415 \| \| 0.007595 \| \| 0.004097 \| \| 0.01389 \| \| 0.003594 \| \| 0.007755 \| \| 0.003947 \| \| 0.003024 \| \| 0.007255 \| \| 0.004616 \| \| 0.005 \| \| 0.01172 \| \| 0.005785 \| \| 0.005665 \| \| 0.002934 \| \| 0.02087 \| \| 0.007723 \| \| 0.00875 \| \| 0.02005 \| \| 0.007736 \| \| 0.004461 \| \| \| --- \| --- \| --- \| --- \| --- \| --- \| --- \| --- \| --- \| --- \| --- \| --- \| --- \| --- \| --- \| --- \| --- \| --- \| --- \| --- \| --- \| --- \| --- \| --- \| --- \| --- \| --- \| --- \| --- \| --- \| --- \| --- \| --- \| --- \| --- \| --- \| --- \| --- \| \|  \| \|  \| \|  \| \|  \| \|  \| \|  \| \|  \| \|  \| \|  \| \|  \| \|  \| \|  \| \|  \| \|  \| \|  \| \|  \| \|  \| \|  \| \|  \| \|  \| \|  \| \|  \| \|  \| \|  \| \|  \| \|  \| \|  \| \|  \| \|  \| \|  \| \|  \| \|  \| \|  \| \|  \| \|  \| \|  \| | \| \| 0.05114 \| \| --- \| \| 0.002869 \| \| 0.03297 \| \| 0.005095 \| \| 0.004399 \| \| 0.002412 \| \| 0.02149 \| \| 0.001974 \| \| 0.002566 \| \| 0.003766 \| \| 0.002348 \| \| 0.003149 \| \| 0.007359 \| \| 0.005329 \| \| 0.01158 \| \| 0.006748 \| \| 0.006245 \| \| 0.006616 \| \| 0.004371 \| \| 0.002875 \| \| 0.0061 \| \| 0.00519 \| \| 0.01138 \| \| 0.004221 \| \| 0.003013 \| \| 0.01212 \| \| 0.003438 \| \| 0.004192 \| \| 0.00286 \| \| 0.002219 \| \| 0.002063 \| \| 0.002637 \| \| 0.003167 \| \| 0.003524 \| \| 0.002532 \| \| 0.002323 \| \| 0.002743 \| \| \| --- \| --- \| --- \| --- \| --- \| --- \| --- \| --- \| --- \| --- \| --- \| --- \| --- \| --- \| --- \| --- \| --- \| --- \| --- \| --- \| --- \| --- \| --- \| --- \| --- \| --- \| --- \| --- \| --- \| --- \| --- \| --- \| --- \| --- \| --- \| --- \| --- \| --- \| \|  \| \|  \| \|  \| \|  \| \|  \| \|  \| \|  \| \|  \| \|  \| \|  \| \|  \| \|  \| \|  \| \|  \| \|  \| \|  \| \|  \| \|  \| \|  \| \|  \| \|  \| \|  \| \|  \| \|  \| \|  \| \|  \| \|  \| \|  \| \|  \| \|  \| \|  \| \|  \| \|  \| \|  \| \|  \| \|  \| | \| 0.001696 \| \| --- \| \| 0.001208 \| \| 0.001151 \| \| 0.001246 \| \| 0.001027 \| \| 0.001656 \| \| 0.001199 \| \| 0.0008384 \| \| 0.002497 \| \| 0.001134 \| \| 0.001423 \| \| 0.001832 \| \| 0.002306 \| \| 0.002626 \| \| 0.003201 \| \| 0.003282 \| \| 0.00112 \| \| 0.004005 \| \| 0.007937 \| \| 0.0009339 \| \| 0.001125 \| \| 0.001069 \| \| 0.001018 \| \| 0.001655 \| \| 0.0006895 \| \| 0.001784 \| \| 0.002328 \| \| 0.005084 \| | \| 0.003465 \| \| --- \| \| 0.002828 \| \| 0.005988 \| \| 0.004415 \| \| 0.00404 \| \| 0.004453 \| \| 0.006327 \| \| 0.002611 \| \| 0.003992 \| \| 0.004767 \| \| 0.002898 \| \| 0.004319 \| \| 0.004045 \| \| 0.003906 \| \| 0.0035 \| \| 0.00415 \| \| 0.006324 \| \| 0.004778 \| \| 0.003908 \| \| 0.001527 \| \| 0.002812 \| \| 0.003263 \| \| 0.00208 \| \| 0.00283 \| \| 0.002581 \| \| 0.002377 \| \| 0.0035 \| \| 0.004676 \| | \| 0.001946 \| \| --- \| \| 0.002662 \| \| 0.002009 \| \| 0.001085 \| \| 0.0018 \| \| 0.003044 \| \| 0.001633 \| \| 0.001267 \| \| 0.00088 \| \| 0.001248 \| \| 0.000871 \| \| 0.000766 \| | \| 0.000908 \| \| --- \| \| 0.002313 \| \| 0.001587 \| \| 0.001239 \| \| 0.001364 \| \| 0.001628 \| \| 0.002347 \| \| 0.000773 \| \| 0.001172 \| \| 0.000461 \| \| 0.003188 \| \| 0.001058 \| |

Concentrations (M)

| EGFP-(s)-CENP-W/mCherry-(s)-CENP-X | EGFP-(s)-CENP-S  mCherry-(s)-CENP-W | EGFP-(s)-CENP-T^ΔC^  mCherry-(s)-CENP-W |
| --- | --- | --- |
| Concentrations (R) (G) (RG) | Concentrations (R) (G) (RG) | Concentrations (R) (G) (RG) |
| \| 6.04E-07 \| 4.36E-07 \| 8.68E-10 \| \| --- \| --- \| --- \| \| 7.03E-07 \| 1.39E-06 \| 5.49E-08 \| \| 4.36E-07 \| 1.15E-06 \| 1.08E-07 \| \| 4.29E-07 \| 7.17E-07 \| 3.32E-08 \| \| 6.93E-07 \| 6.01E-07 \| 8.97E-09 \| \| 6.49E-07 \| 4.68E-07 \| 8.13E-09 \| \| 4.57E-07 \| 5.14E-07 \| 2.75E-08 \| \| 1.14E-06 \| 2.94E-07 \| 1.81E-08 \| \| 9.57E-07 \| 4.89E-07 \| 3.57E-08 \| \| 5.34E-07 \| 4.92E-07 \| 5.40E-08 \| \| 5.36E-07 \| 2.84E-07 \| 5.29E-09 \| | \| 2.22E-07 \| 4.07E-07 \| 4.45E-08 \| \| --- \| --- \| --- \| \| 3.43E-07 \| 1.60E-07 \| 6.33E-09 \| \| 2.96E-07 \| 1.38E-07 \| 7.50E-09 \| \| 1.69E-07 \| 1.48E-07 \| 1.59E-08 \| \| 1.72E-07 \| 1.17E-07 \| 2.24E-08 \| \| 1.67E-07 \| 1.54E-07 \| 5.85E-08 \| \| 1.22E-07 \| 6.21E-08 \| 1.73E-08 \| \| 3.59E-07 \| 7.24E-07 \| 6.04E-08 \| \| 4.39E-07 \| 8.27E-07 \| 8.04E-09 \| \| 4.89E-07 \| 4.99E-07 \| 3.48E-08 \| \| 2.80E-07 \| 2.25E-07 \| 1.62E-08 \| \| 3.98E-07 \| 4.51E-07 \| 3.60E-08 \| | \| 3.74E-07 \| 2.64E-07 \| 4.26E-09 \| \| --- \| --- \| --- \| \| 5.83E-07 \| 2.32E-07 \| 1.60E-08 \| \| 5.39E-07 \| 2.81E-07 \| 2.00E-08 \| \| 2.30E-07 \| 1.17E-07 \| 1.43E-08 \| \| 2.22E-07 \| 1.53E-07 \| 5.18E-09 \| \| 3.81E-07 \| 7.58E-07 \| 1.31E-07 \| \| 2.67E-07 \| 8.21E-08 \| 4.20E-09 \| \| 1.72E-07 \| 2.28E-07 \| 2.02E-08 \| \| 1.40E-07 \| 1.11E-07 \| 2.73E-08 \| \| 7.66E-08 \| 1.76E-07 \| 1.04E-08 \| \| 2.25E-07 \| 9.86E-08 \| 2.44E-08 \| \| 1.34E-07 \| 9.17E-08 \| 6.56E-09 \| \| 8.29E-08 \| 5.38E-08 \| 6.48E-09 \| \| 3.73E-07 \| 2.07E-07 \| 4.69E-08 \| \| 3.66E-07 \| 1.43E-07 \| 6.34E-08 \| \| 4.10E-07 \| 2.10E-07 \| 8.73E-09 \| \| 3.47E-07 \| 2.86E-07 \| 7.43E-09 \| \| 2.83E-07 \| 4.42E-07 \| 3.39E-08 \| \| 3.68E-07 \| 3.71E-07 \| 8.01E-08 \| \| 5.18E-07 \| 3.36E-07 \| 2.15E-08 \| \| 2.85E-07 \| 3.10E-07 \| 2.10E-10 \| \| 7.06E-07 \| 3.85E-07 \| 2.31E-08 \| \| 5.27E-07 \| 7.91E-07 \| 1.08E-07 \| \|  \|  \|  \| \|  \|  \|  \| \|  \|  \|  \| \|  \|  \|  \| \|  \|  \|  \| \|  \|  \|  \| \|  \|  \|  \| \|  \|  \|  \| \|  \|  \|  \| \|  \|  \|  \| \|  \|  \|  \| \|  \|  \|  \| \|  \|  \|  \| \|  \|  \|  \| \|  \|  \|  \| \|  \|  \|  \| \|  \|  \|  \| \|  \|  \|  \| \|  \|  \|  \| \|  \|  \|  \| \|  \|  \|  \| \|  \|  \|  \| \|  \|  \|  \| |

| EGFP-(s)-Spc25/mCherry-(s)-CENP-T^ΔC^ | EGFP-(s)-Spc24/mCherry-(s)-CENPT^ΔN^ | mCherry-(s)-CENP-T/EGFP-(s)-CENP-X |
| --- | --- | --- |
| \| 7.54E-07 \| 7.25E-07 \| 1.41E-07 \| \| --- \| --- \| --- \| \| 3.30E-07 \| 3.45E-07 \| 7.87E-08 \| \| 2.21E-07 \| 1.01E-07 \| 1.44E-10 \| \| 1.01E-06 \| 4.82E-07 \| 1.32E-07 \| \| 5.62E-07 \| 1.07E-06 \| 1.25E-07 \| \| 6.55E-07 \| 7.73E-07 \| 9.85E-09 \| \| 1.87E-0 \| 6.56E-07 \| 3.44E-08 \| \| 2.87E-07 \| 2.04E-07 \| 5.27E-09 \| \| 1.31E-07 \| 1.69E-07 \| 2.42E-08 \| \| 1.27E-07 \| 2.48E-07 \| 1.94E-08 \| \| 1.02E-07 \| 1.63E-07 \| 7.07E-09 \| \| 8.36E-08 \| 1.83E-07 \| 4.82E-09 \| \| 3.32E-07 \| 7.29E-07 \| 2.39E-08 \| \| 4.51E-07 \| 8.76E-07 \| 6.66E-08 \| \| 7.39E-07 \| 5.00E-07 \| 2.25E-09 \| | \| 2.52E-07 \| 3.86E-07 \| 1.01E-08 \| \| --- \| --- \| --- \| \| 5.04E-07 \| 3.36E-07 \| 6.47E-09 \| \| 3.27E-07 \| 2.26E-07 \| 1.01E-09 \| \| 2.21E-07 \| 3.74E-07 \| 1.07E-07 \| \| 2.74E-07 \| 3.28E-07 \| 1.88E-08 \| \| 3.44E-07 \| 2.68E-07 \| 1.52E-07 \| \| 2.73E-07 \| 6.83E-07 \| 6.79E-09 \| \| 2.19E-07 \| 4.71E-07 \| 5.71E-09 \| \| 1.12E-07 \| 5.94E-08 \| 4.22E-09 \| \| 3.80E-07 \| 2.48E-07 \| 3.45E-08 \| \| 4.34E-07 \| 3.51E-07 \| 1.82E-08 \| \| 3.34E-07 \| 2.74E-07 \| 4.85E-08 \| \| 5.21E-07 \| 7.37E-07 \| 2.60E-09 \| \| 5.68E-07 \| 7.75E-07 \| 1.33E-07 \| \|  \|  \|  \| \|  \|  \|  \| \|  \|  \|  \| \|  \|  \|  \| \|  \|  \|  \| \|  \|  \|  \| \|  \|  \|  \| | \| 1.84E-06 \| 7.24E-07 \| 2.33E-07 \| \| --- \| --- \| --- \| \| 1.34E-07 \| 5.47E-08 \| 9.00E-09 \| \| 4.74E-07 \| 1.57E-07 \| 1.44E-08 \| \| 1.68E-07 \| 9.23E-08 \| 1.80E-09 \| \| 9.99E-08 \| 6.20E-08 \| 1.84E-08 \| \| 1.04E-07 \| 7.00E-08 \| 2.12E-09 \| \| 1.04E-07 \| 4.16E-08 \| 4.35E-09 \| |

| EGFP-(s)-CENP-S/mCherry-(s)-CENP-X | EGFP-(s)-CENP-W/mCherry-(s)-CENP-T | EGFP-(s)-CENP-T^ΔN^/mCherry-(s)-CENP-W |
| --- | --- | --- |
| Concentrations (R) (G) (RG) | Concentrations (R) (G) (RG) | Concentrations (R) (G) (RG) |
| \| 3.75E-07 \| 6.19E-08 \| 7.57E-08 \| \| --- \| --- \| --- \| \| 1.91E-07 \| 2.16E-07 \| 1.35E-07 \| \| 1.01E-07 \| 8.00E-08 \| 3.58E-08 \| \| 1.32E-07 \| 5.37E-08 \| 4.60E-08 \| \| 1.34E-07 \| 2.35E-08 \| 2.15E-08 \| \| 5.90E-09 \| 6.06E-08 \| 3.51E-08 \| \| 1.25E-08 \| 3.73E-08 \| 2.51E-08 \| \| 2.84E-09 \| 5.74E-09 \| 9.31E-09 \| \| 2.63E-08 \| 1.11E-09 \| 7.45E-09 \| \| 4.04E-08 \| 1.93E-08 \| 1.31E-08 \| \| 2.62E-07 \| 3.81E-08 \| 3.53E-08 \| \| 5.46E-09 \| 6.29E-09 \| 6.22E-09 \| \| 4.06E-07 \| 1.10E-06 \| 3.78E-07 \| \| 8.45E-07 \| 3.95E-07 \| 5.98E-08 \| \| 7.12E-07 \| 9.96E-07 \| 6.53E-07 \| \| 1.38E-07 \| 1.39E-06 \| 3.62E-07 \| \| 6.77E-07 \| 1.45E-07 \| 1.23E-06 \| \| 3.21E-07 \| 1.09E-06 \| 8.42E-08 \| \| 4.47E-07 \| 2.42E-07 \| 2.46E-08 \| \| 3.22E-07 \| 2.02E-07 \| 1.29E-07 \| \| 3.54E-07 \| 3.95E-07 \| 3.33E-08 \| \| 4.83E-07 \| 6.71E-07 \| 2.74E-07 \| \| 2.15E-07 \| 1.71E-06 \| 4.00E-07 \| \| 7.74E-07 \| 4.99E-07 \| 3.19E-07 \| \| 6.31E-07 \| 3.39E-07 \| 4.51E-07 \| \| 7.73E-07 \| 6.25E-07 \| 1.06E-07 \| \| 3.15E-07 \| 1.23E-06 \| 6.45E-07 \| \| 2.25E-07 \| 1.09E-06 \| 5.87E-07 \| \| 5.38E-07 \| 1.01E-06 \| 3.30E-07 \| \| 2.56E-07 \| 3.89E-07 \| 1.06E-06 \| \| 5.50E-07 \| 7.17E-07 \| 5.86E-07 \| \| 5.70E-07 \| 1.31E-06 \| 2.97E-07 \| \| 4.96E-09 \| 5.34E-07 \| 1.35E-07 \| \| 1.51E-07 \| 9.66E-08 \| 1.48E-08 \| \| 1.41E-07 \| 7.26E-08 \| 6.97E-09 \| \| 7.00E-07 \| 1.12E-06 \| 6.55E-07 \| \| 1.00E-06 \| 6.76E-07 \| 1.11E-07 \| \| 6.72E-07 \| 2.49E-07 \| 4.92E-07 \| \| 1.65E-06 \| 2.76E-07 \| 2.58E-07 \| \| 1.36E-06 \| 3.74E-07 \| 3.51E-07 \| \| 5.02E-07 \| 3.47E-07 \| 6.25E-07 \| \| 1.19E-06 \| 2.13E-06 \| 4.39E-07 \| \| 6.93E-07 \| 1.73E-06 \| 1.04E-06 \| \| 9.42E-07 \| 1.16E-06 \| 5.60E-07 \| \| 1.31E-06 \| 1.03E-06 \| 4.25E-07 \| \| 1.93E-06 \| 7.06E-07 \| 1.32E-06 \| \| 1.70E-07 \| 9.97E-07 \| 2.27E-07 \| \| 1.14E-06 \| 1.29E-06 \| 2.41E-07 \| \| 2.71E-06 \| 9.12E-07 \| 1.46E-07 \| \| 4.02E-07 \| 7.02E-07 \| 3.91E-08 \| \| 4.27E-07 \| 3.66E-07 \| 1.08E-07 \| \| 3.69E-07 \| 3.65E-07 \| 5.41E-08 \| \| 1.09E-06 \| 6.71E-07 \| 4.61E-07 \| \| 4.08E-07 \| 1.67E-06 \| 3.67E-07 \| \| 7.95E-07 \| 1.38E-06 \| 4.20E-07 \| \| 7.62E-07 \| 1.55E-06 \| 3.40E-07 \| \| 7.30E-07 \| 1.58E-06 \| 7.74E-07 \| \| 2.77E-07 \| 2.02E-06 \| 7.84E-07 \| \| 6.93E-07 \| 1.61E-06 \| 3.92E-07 \| \| 4.65E-07 \| 1.52E-07 \| 1.44E-07 \| \| 6.12E-07 \| 2.10E-07 \| 6.66E-08 \| \| 5.89E-07 \| 1.71E-07 \| 4.03E-08 \| \| 4.81E-07 \| 1.56E-07 \| 1.11E-07 \| \| 4.34E-07 \| 3.61E-07 \| 2.19E-07 \| \| 8.04E-07 \| 4.39E-07 \| 5.19E-07 \| \| 4.02E-07 \| 1.61E-07 \| 1.52E-07 \| \| 1.99E-07 \| 2.18E-07 \| 1.47E-07 \| \| 1.49E-07 \| 1.86E-07 \| 8.01E-08 \| \| 1.25E-07 \| 1.97E-07 \| 1.01E-07 \| \| 8.22E-08 \| 2.74E-07 \| 1.04E-07 \| \| 3.86E-07 \| 1.54E-07 \| 1.11E-07 \| \| 6.41E-07 \| 1.52E-07 \| 3.88E-07 \| \| 6.97E-07 \| 1.55E-07 \| 2.10E-07 \| \| 2.61E-07 \| 1.73E-07 \| 8.15E-08 \| \| 1.85E-07 \| 2.41E-07 \| 1.52E-07 \| \| 3.92E-07 \| 5.26E-07 \| 1.69E-07 \| \| 4.58E-07 \| 6.10E-07 \| 5.08E-07 \| \| 4.88E-07 \| 1.35E-07 \| 2.77E-07 \| \| 3.16E-07 \| 2.47E-07 \| 1.79E-07 \| \| 3.86E-07 \| 1.72E-07 \| 2.17E-07 \| \| 3.03E-07 \| 1.95E-07 \| 1.58E-07 \| \| 1.98E-07 \| 1.34E-07 \| 1.34E-07 \| \| 3.49E-07 \| 2.26E-07 \| 1.63E-07 \| \| 2.90E-07 \| 2.42E-07 \| 1.56E-07 \| \| 2.78E-07 \| 2.10E-07 \| 1.49E-07 \| \| 3.78E-07 \| 2.58E-07 \| 2.22E-07 \| \| 3.72E-07 \| 2.26E-07 \| 1.71E-07 \| \| 3.87E-07 \| 1.80E-07 \| 1.74E-07 \| \| 7.47E-07 \| 1.93E-07 \| 2.36E-07 \| \| 8.48E-07 \| 2.02E-07 \| 2.37E-07 \| \| 2.76E-07 \| 4.21E-07 \| 7.63E-08 \| \| 2.35E-07 \| 2.87E-07 \| 1.56E-07 \| \| 2.64E-07 \| 4.51E-07 \| 1.80E-07 \| \| 2.37E-07 \| 9.03E-08 \| 6.17E-08 \| \| 2.62E-07 \| 4.61E-08 \| 5.27E-08 \| \| 5.95E-07 \| 4.59E-07 \| 1.57E-07 \| \| 2.12E-06 \| 2.11E-06 \| 2.10E-06 \| \| 1.87E-06 \| 1.15E-06 \| 9.46E-07 \| \| 2.20E-06 \| 1.43E-06 \| 1.31E-06 \| \| 1.37E-06 \| 2.62E-06 \| 1.73E-06 \| \| 6.68E-07 \| 5.54E-07 \| 2.93E-07 \| | \| 2.64E-07 \| 3.05E-07 \| 1.19E-07 \| \| --- \| --- \| --- \| \| 4.21E-07 \| 2.67E-07 \| 4.04E-08 \| \| 4.61E-07 \| 2.00E-07 \| 8.48E-08 \| \| 8.41E-07 \| 1.35E-06 \| 4.28E-08 \| \| 8.37E-07 \| 1.17E-06 \| 5.69E-08 \| \| 7.15E-07 \| 1.02E-06 \| 1.15E-07 \| \| 5.16E-07 \| 1.83E-07 \| 8.84E-08 \| \| 4.68E-07 \| 1.41E-07 \| 6.09E-08 \| \| 3.70E-07 \| 1.64E-07 \| 5.00E-08 \| \| 3.04E-07 \| 1.81E-07 \| 6.45E-08 \| \| 4.27E-07 \| 1.51E-07 \| 7.82E-08 \| \| 2.43E-07 \| 9.17E-08 \| 3.58E-07 \| \| 4.94E-07 \| 1.22E-07 \| 1.20E-07 \| \| 4.01E-07 \| 1.41E-07 \| 1.02E-07 \| \| 3.12E-07 \| 1.28E-07 \| 5.62E-08 \| \| 3.28E-07 \| 1.54E-07 \| 1.63E-08 \| \| 4.39E-07 \| 9.34E-08 \| 1.06E-07 \| \| 9.01E-08 \| 9.09E-08 \| 1.22E-07 \| \| 1.94E-07 \| 1.49E-07 \| 1.75E-07 \| \| 2.12E-07 \| 1.25E-07 \| 1.56E-07 \| \| 3.83E-07 \| 1.39E-07 \| 3.17E-07 \| \| 4.32E-07 \| 1.52E-07 \| 1.96E-07 \| \| 2.17E-07 \| 1.67E-07 \| 3.78E-08 \| \| 2.09E-07 \| 2.01E-07 \| 7.46E-08 \| \| 1.85E-07 \| 1.64E-07 \| 7.86E-08 \| \| 1.72E-07 \| 1.51E-07 \| 5.74E-08 \| \| 1.96E-07 \| 1.36E-07 \| 4.29E-08 \| \| 2.30E-06 \| 1.02E-06 \| 9.47E-07 \| \| 2.72E-06 \| 9.69E-07 \| 6.71E-07 \| \| 4.27E-06 \| 9.49E-07 \| 3.04E-07 \| \| 2.37E-06 \| 4.65E-07 \| 7.56E-07 \| \| 1.48E-06 \| 5.91E-07 \| 6.53E-07 \| \| 2.36E-06 \| 3.62E-07 \| 7.23E-08 \| \| 4.35E-06 \| 6.90E-07 \| 5.44E-07 \| \| 2.79E-06 \| 7.56E-07 \| 7.65E-07 \| \| 2.71E-06 \| 5.79E-07 \| 9.22E-07 \| \| 5.07E-07 \| 2.48E-06 \| 9.12E-07 \| \| 2.21E-06 \| 1.06E-06 \| 1.63E-06 \| \| 1.32E-06 \| 2.20E-06 \| 1.57E-07 \| \| 1.55E-06 \| 1.87E-06 \| 4.47E-08 \| \| 5.88E-07 \| 4.26E-07 \| 1.36E-06 \| \| 8.81E-07 \| 1.11E-06 \| 4.07E-07 \| \| 1.18E-06 \| 1.31E-06 \| 1.16E-07 \| \| 9.32E-07 \| 2.13E-06 \| 1.55E-08 \| \| 9.41E-07 \| 1.20E-06 \| 7.77E-08 \| \| 1.03E-06 \| 1.11E-06 \| 5.19E-09 \| \| 1.12E-06 \| 9.59E-07 \| 3.59E-07 \| \| 9.71E-07 \| 6.86E-07 \| 2.12E-07 \| \| 1.57E-06 \| 9.65E-07 \| 6.65E-07 \| \| 1.06E-06 \| 7.88E-07 \| 8.77E-07 \| \| 2.11E-06 \| 1.01E-06 \| 7.93E-07 \| \| 1.95E-06 \| 6.35E-07 \| 1.37E-06 \| \| 1.76E-06 \| 6.06E-07 \| 1.17E-06 \| \| 2.78E-06 \| 1.11E-06 \| 4.97E-07 \| \| 3.06E-06 \| 7.30E-07 \| 2.29E-07 \| \| 2.74E-06 \| 8.40E-07 \| 3.94E-07 \| \| 6.61E-07 \| 2.85E-07 \| 2.48E-08 \| \| 5.32E-07 \| 2.03E-07 \| 1.26E-07 \| \| 4.71E-07 \| 1.83E-07 \| 1.07E-07 \| \| 5.27E-07 \| 5.08E-07 \| 1.96E-07 \| \| 6.89E-07 \| 2.35E-07 \| 2.14E-07 \| \| 1.57E-07 \| 1.69E-07 \| 3.77E-08 \| \| 1.65E-07 \| 1.82E-07 \| 6.24E-08 \| \| 1.56E-07 \| 1.35E-07 \| 8.47E-08 \| \| 2.50E-07 \| 2.99E-07 \| 1.32E-07 \| \| 4.65E-07 \| 1.33E-06 \| 1.49E-07 \| \| 2.62E-07 \| 1.64E-07 \| 7.75E-08 \| \| 1.60E-07 \| 1.22E-07 \| 1.28E-07 \| \| 1.54E-07 \| 9.56E-08 \| 1.03E-07 \| \| 1.28E-07 \| 8.36E-08 \| 9.20E-08 \| \| 1.85E-07 \| 7.97E-08 \| 7.78E-08 \| \| 5.05E-08 \| 8.94E-08 \| 1.09E-07 \| \| 1.44E-07 \| 2.11E-07 \| 1.26E-07 \| \| 3.91E-07 \| 1.02E-07 \| 1.07E-07 \| \| 5.12E-07 \| 9.95E-08 \| 1.14E-07 \| \| 1.86E-06 \| 3.84E-07 \| 3.31E-07 \| \| 6.32E-07 \| 1.02E-07 \| 8.41E-08 \| \| 1.29E-06 \| 4.64E-07 \| 4.60E-07 \| \| 1.17E-06 \| 1.30E-06 \| 7.69E-07 \| \| 2.58E-07 \| 7.97E-07 \| 1.50E-07 \| \| 3.69E-07 \| 2.65E-07 \| 1.20E-07 \| \| 1.19E-07 \| 8.33E-08 \| 1.81E-08 \| \| 1.09E-07 \| 6.85E-08 \| 2.68E-08 \| \| 2.73E-07 \| 7.01E-08 \| 5.20E-08 \| \| 2.70E-07 \| 1.72E-07 \| 7.65E-08 \| \| 1.21E-07 \| 1.99E-07 \| 5.51E-08 \| \| 1.11E-07 \| 1.75E-07 \| 4.07E-08 \| \| 1.19E-07 \| 1.48E-07 \| 4.09E-08 \| \| 9.67E-08 \| 8.74E-08 \| 2.34E-08 \| \| 9.44E-08 \| 5.30E-08 \| 1.66E-08 \| \| 9.72E-08 \| 1.12E-07 \| 2.40E-08 \| \| 1.10E-07 \| 9.26E-08 \| 3.11E-08 \| \| 5.16E-07 \| 3.41E-07 \| 1.02E-07 \| \| 6.11E-07 \| 4.40E-07 \| 1.14E-07 \| \| 5.58E-07 \| 4.57E-07 \| 1.21E-07 \| \| 4.27E-07 \| 1.17E-06 \| 2.34E-07 \| \| 6.92E-08 \| 8.53E-08 \| 3.62E-08 \| \| 5.69E-08 \| 5.18E-08 \| 1.02E-08 \| \| 4.84E-08 \| 3.75E-08 \| 1.38E-08 \| \| 5.34E-08 \| 2.94E-08 \| 1.53E-08 \| \| 3.80E-08 \| 3.30E-08 \| 1.47E-08 \| \| 4.00E-08 \| 3.30E-08 \| 1.43E-08 \| \| 4.92E-08 \| 3.62E-08 \| 1.86E-08 \| \| 7.99E-08 \| 1.15E-07 \| 9.46E-08 \| \| 1.09E-07 \| 7.73E-08 \| 5.57E-08 \| \| 7.38E-08 \| 7.11E-08 \| 8.91E-08 \| \| 9.07E-08 \| 6.20E-08 \| 6.17E-08 \| \| 8.83E-08 \| 5.83E-08 \| 5.24E-08 \| \| 6.21E-08 \| 7.50E-08 \| 7.16E-08 \| \| 2.75E-07 \| 2.56E-07 \| 3.15E-07 \| \| 4.03E-07 \| 2.48E-07 \| 1.31E-07 \| \| 1.31E-06 \| 2.25E-07 \| 1.98E-07 \| \| 1.15E-06 \| 1.97E-07 \| 2.06E-07 \| \| 6.17E-07 \| 8.11E-07 \| 7.94E-07 \| \| 1.06E-06 \| 5.93E-07 \| 5.78E-07 \| \| 1.23E-06 \| 5.44E-07 \| 5.94E-07 \| \| 1.31E-06 \| 5.79E-07 \| 7.43E-07 \| \| 1.45E-06 \| 5.12E-07 \| 3.87E-07 \| \| 1.03E-06 \| 4.73E-07 \| 3.13E-07 \| \| 1.25E-06 \| 4.07E-07 \| 4.03E-07 \| \| 1.31E-06 \| 3.27E-07 \| 1.81E-07 \| \| 1.32E-06 \| 1.61E-06 \| 5.52E-07 \| \| 1.14E-06 \| 1.33E-06 \| 3.74E-07 \| \| 1.14E-06 \| 7.89E-07 \| 3.85E-07 \| \| 1.23E-06 \| 5.92E-07 \| 3.44E-07 \| \| 2.70E-07 \| 4.62E-07 \| 1.06E-07 \| \| 2.14E-07 \| 3.75E-07 \| 1.23E-07 \| \| 2.34E-07 \| 3.54E-07 \| 8.02E-08 \| \| 7.60E-07 \| 1.25E-06 \| 8.50E-07 \| \| 1.00E-06 \| 9.77E-07 \| 6.28E-07 \| \| 1.07E-06 \| 1.07E-06 \| 6.87E-07 \| \| 9.66E-07 \| 8.82E-07 \| 5.74E-07 \| \| 7.96E-07 \| 7.60E-07 \| 6.29E-07 \| \| 8.64E-07 \| 8.39E-07 \| 6.75E-07 \| \| 4.74E-07 \| 5.80E-07 \| 5.44E-07 \| \| 4.62E-07 \| 5.27E-07 \| 2.08E-07 \| \| 2.55E-07 \| 2.68E-07 \| 1.08E-07 \| \| 2.65E-07 \| 2.68E-07 \| 1.07E-07 \| \| 2.88E-07 \| 3.06E-07 \| 1.21E-07 \| \| 1.99E-07 \| 4.63E-07 \| 2.17E-07 \| \| 3.72E-07 \| 7.47E-07 \| 2.66E-07 \| \| 4.62E-07 \| 4.87E-07 \| 2.58E-07 \| \| 1.37E-07 \| 2.98E-07 \| 8.11E-08 \| \| 3.02E-08 \| 4.43E-08 \| 4.34E-09 \| \| 4.24E-08 \| 2.35E-08 \| 9.81E-09 \| \| 2.37E-08 \| 3.13E-08 \| 2.95E-09 \| \| 2.60E-08 \| 3.03E-08 \| 7.66E-09 \| \| 1.62E-08 \| 2.78E-08 \| 9.56E-09 \| \| 1.84E-08 \| 2.18E-08 \| 1.14E-08 \| \| 1.84E-08 \| 5.50E-08 \| 1.65E-08 \| \| 1.24E-08 \| 3.02E-08 \| 7.24E-09 \| \| 1.77E-08 \| 2.19E-08 \| 7.02E-09 \| \| 2.34E-06 \| 9.37E-07 \| 1.22E-06 \| \| 2.70E-06 \| 8.85E-07 \| 1.20E-06 \| \| 2.70E-06 \| 5.76E-07 \| 8.11E-07 \| \| 2.53E-06 \| 5.90E-07 \| 7.01E-07 \| \| 2.01E-07 \| 1.34E-07 \| 1.09E-07 \| \| 2.16E-07 \| 1.24E-07 \| 8.93E-08 \| \| 2.07E-07 \| 8.78E-08 \| 5.92E-08 \| \| 2.26E-07 \| 5.55E-08 \| 7.49E-08 \| \| 2.89E-07 \| 4.56E-08 \| 5.61E-08 \| \| 3.28E-07 \| 6.53E-08 \| 5.93E-08 \| \| 2.42E-07 \| 9.61E-08 \| 5.71E-08 \| \| 5.35E-07 \| 1.44E-07 \| 1.21E-07 \| \| 2.77E-07 \| 1.16E-07 \| 7.56E-08 \| \| 9.44E-07 \| 3.88E-07 \| 3.00E-07 \| \| 4.42E-07 \| 2.27E-07 \| 1.08E-07 \| \| 4.20E-07 \| 1.11E-07 \| 1.15E-07 \| \| 5.47E-07 \| 3.54E-07 \| 3.08E-07 \| \| 2.60E-07 \| 1.07E-07 \| 7.02E-08 \| \| 2.59E-07 \| 1.14E-07 \| 7.40E-08 \| \| 2.15E-07 \| 2.49E-07 \| 1.88E-07 \| \| 2.89E-07 \| 2.06E-07 \| 1.32E-07 \| \| 3.89E-07 \| 9.43E-08 \| 1.35E-07 \| \| 3.07E-07 \| 6.88E-08 \| 1.10E-07 \| \| 4.78E-07 \| 3.15E-07 \| 1.28E-07 \| \| 7.09E-07 \| 8.13E-07 \| 7.33E-07 \| \| 7.84E-07 \| 2.09E-07 \| 1.39E-07 \| \| 8.45E-07 \| 1.99E-07 \| 1.29E-07 \| \| 2.44E-06 \| 3.92E-07 \| 4.65E-07 \| \| 1.78E-07 \| 6.34E-07 \| 1.19E-07 \| \| 2.39E-07 \| 5.18E-07 \| 1.32E-07 \| \| 2.72E-07 \| 4.92E-07 \| 9.69E-08 \| \| 7.93E-07 \| 6.77E-07 \| 3.27E-07 \| \| 9.32E-07 \| 5.63E-07 \| 3.13E-07 \| \| 9.73E-07 \| 4.66E-07 \| 2.66E-07 \| \| 4.58E-07 \| 7.00E-07 \| 2.01E-07 \| \| 4.91E-07 \| 6.45E-07 \| 1.48E-07 \| \| 2.94E-07 \| 8.67E-07 \| 2.62E-07 \| \| 4.56E-07 \| 6.62E-07 \| 1.43E-07 \| \| 4.10E-07 \| 4.67E-07 \| 1.23E-07 \| \| 3.89E-07 \| 4.07E-07 \| 1.12E-07 \| \| 3.95E-07 \| 3.68E-07 \| 1.25E-07 \| \| 1.93E-07 \| 4.99E-07 \| 6.12E-08 \| \| 1.72E-07 \| 1.79E-07 \| 5.99E-08 \| \| 1.78E-07 \| 1.23E-07 \| 3.43E-08 \| \| 3.02E-07 \| 8.54E-08 \| 5.13E-08 \| \| 9.03E-08 \| 1.20E-07 \| 7.52E-08 \| \| 1.11E-07 \| 8.83E-08 \| 5.00E-08 \| \| 1.12E-07 \| 6.06E-08 \| 3.42E-08 \| \| 1.29E-06 \| 7.59E-07 \| 3.21E-07 \| \| 9.47E-07 \| 4.35E-07 \| 3.67E-07 \| \| 2.45E-07 \| 4.12E-07 \| 4.50E-07 \| \| 1.23E-06 \| 6.96E-07 \| 3.59E-07 \| \| 1.30E-06 \| 7.22E-07 \| 3.34E-08 \| \| 1.03E-06 \| 4.92E-07 \| 1.84E-07 \| \| 7.80E-07 \| 3.97E-07 \| 9.35E-08 \| \| 1.88E-07 \| 3.95E-07 \| 2.67E-07 \| \| 3.14E-07 \| 3.59E-07 \| 1.38E-07 \| \| 1.95E-07 \| 3.81E-07 \| 1.53E-07 \| \| 3.76E-07 \| 3.45E-07 \| 1.40E-07 \| \| 2.77E-07 \| 3.06E-07 \| 1.80E-07 \| \| 2.22E-07 \| 2.20E-07 \| 1.24E-07 \| \| 2.82E-07 \| 1.88E-07 \| 9.81E-08 \| \| 2.47E-07 \| 1.97E-07 \| 7.04E-08 \| \| 3.44E-07 \| 1.73E-07 \| 1.44E-07 \| \| 2.35E-07 \| 1.19E-07 \| 1.39E-07 \| \| 2.51E-07 \| 1.48E-07 \| 7.07E-08 \| \| 9.95E-08 \| 9.19E-08 \| 5.32E-08 \| \| 4.37E-07 \| 3.34E-07 \| 4.64E-07 \| \| 4.10E-07 \| 3.81E-07 \| 3.86E-07 \| \| 4.07E-07 \| 3.15E-07 \| 3.58E-07 \| \| 5.05E-07 \| 3.15E-07 \| 4.69E-07 \| \| 5.79E-07 \| 2.38E-07 \| 2.48E-07 \| \| 6.70E-07 \| 2.13E-07 \| 1.47E-07 \| \| 5.12E-07 \| 1.78E-07 \| 1.10E-07 \| \| 4.40E-07 \| 1.44E-07 \| 8.45E-08 \| \| 3.79E-07 \| 1.20E-07 \| 8.82E-08 \| | \| 4.48E-07 \| 4.38E-07 \| 1.08E-07 \| \| --- \| --- \| --- \| \| 4.40E-07 \| 3.22E-07 \| 5.20E-08 \| \| 2.36E-07 \| 6.13E-08 \| 5.77E-08 \| \| 2.57E-07 \| 9.94E-08 \| 2.09E-08 \| \| 1.77E-07 \| 1.65E-07 \| 1.60E-08 \| \| 1.72E-07 \| 1.24E-07 \| 1.79E-08 \| \| 1.90E-07 \| 1.90E-07 \| 7.83E-09 \| \| 1.57E-07 \| 8.39E-08 \| 1.15E-08 \| \| 1.15E-07 \| 1.15E-07 \| 1.49E-08 \| \| 2.84E-08 \| 4.80E-08 \| 1.07E-07 \| \| 1.40E-07 \| 7.35E-08 \| 8.74E-09 \| \| 1.17E-07 \| 1.56E-07 \| 2.23E-08 \| \| 7.67E-08 \| 5.02E-08 \| 2.11E-08 \| \| 8.54E-07 \| 5.73E-07 \| 9.66E-08 \| \| 1.30E-07 \| 1.14E-07 \| 2.43E-07 \| \| 3.58E-07 \| 2.64E-07 \| 2.39E-08 \| \| 2.42E-07 \| 2.31E-07 \| 2.14E-08 \| \| 1.92E-07 \| 2.25E-07 \| 5.43E-08 \| \| 2.78E-07 \| 1.51E-07 \| 1.11E-08 \| \| 2.42E-07 \| 1.20E-07 \| 2.32E-08 \| \| 1.55E-07 \| 1.07E-07 \| 9.80E-09 \| \| 1.10E-07 \| 1.18E-07 \| 1.52E-08 \| \| 9.68E-08 \| 6.98E-08 \| 5.36E-09 \| \| 1.01E-07 \| 1.13E-07 \| 4.03E-08 \| \| 1.70E-07 \| 1.76E-07 \| 5.24E-08 \| \| 1.42E-07 \| 2.93E-07 \| 1.71E-08 \| \| 1.03E-07 \| 2.14E-07 \| 8.05E-09 \| \| 4.47E-07 \| 1.41E-07 \| 7.97E-08 \| \| 3.44E-07 \| 1.45E-07 \| 5.01E-08 \| \| 2.75E-07 \| 1.74E-07 \| 2.91E-08 \| \| 9.16E-07 \| 7.59E-07 \| 2.77E-07 \| \| 1.11E-06 \| 8.60E-07 \| 2.10E-07 \| \| 1.10E-06 \| 6.10E-07 \| 2.53E-07 \| \| 7.85E-07 \| 3.98E-07 \| 1.12E-07 \| \| 1.17E-06 \| 5.74E-07 \| 1.29E-07 \| \| 5.72E-08 \| 5.25E-08 \| 3.71E-09 \| \| 6.31E-08 \| 5.48E-08 \| 8.60E-09 \| \| 6.37E-08 \| 5.14E-08 \| 1.26E-08 \| \| 5.76E-08 \| 3.38E-08 \| 2.81E-08 \| \| 8.76E-08 \| 4.41E-08 \| 1.97E-08 \| \| 7.86E-08 \| 4.00E-08 \| 2.92E-08 \| \| 5.28E-08 \| 3.46E-08 \| 1.05E-08 \| \| 1.83E-07 \| 3.96E-08 \| 1.38E-08 \| \| 5.18E-08 \| 2.86E-08 \| 3.09E-09 \| \| 6.86E-08 \| 7.13E-08 \| 2.88E-08 \| \| 7.57E-08 \| 5.44E-08 \| 6.67E-09 \| \| 1.45E-07 \| 5.24E-08 \| 8.56E-09 \| \| 9.42E-09 \| 1.20E-08 \| 2.87E-08 \| \| 2.39E-08 \| 2.21E-08 \| 2.79E-09 \| \| 2.50E-08 \| 1.63E-08 \| 1.51E-09 \| \| 1.49E-08 \| 6.84E-09 \| 5.97E-10 \| \| 3.68E-08 \| 2.10E-08 \| 8.26E-09 \| \| 1.31E-07 \| 2.01E-07 \| 1.13E-08 \| \| 1.23E-07 \| 2.04E-07 \| 6.01E-09 \| \| 1.24E-07 \| 1.65E-07 \| 8.70E-09 \| \| 7.27E-07 \| 7.48E-07 \| 1.73E-07 \| \| 1.41E-07 \| 9.92E-08 \| 6.35E-09 \| \| 1.46E-07 \| 8.59E-08 \| 6.45E-09 \| \| 1.01E-07 \| 9.16E-08 \| 3.94E-09 \| \| 1.13E-07 \| 6.29E-08 \| 1.03E-08 \| \| 1.33E-07 \| 5.87E-08 \| 4.83E-09 \| \| 9.16E-08 \| 1.58E-07 \| 1.01E-08 \| \| 9.36E-08 \| 1.09E-07 \| 9.79E-10 \| \| 8.79E-08 \| 5.23E-08 \| 4.91E-09 \| \| 7.57E-08 \| 7.02E-08 \| 6.14E-09 \| \| 4.13E-07 \| 3.76E-07 \| 1.16E-07 \| \| 3.97E-07 \| 2.72E-07 \| 9.29E-08 \| \| 4.30E-07 \| 2.28E-07 \| 2.92E-08 \| \| 3.57E-07 \| 2.55E-07 \| 4.27E-08 \| \| 3.46E-07 \| 2.02E-07 \| 1.96E-08 \| \| 7.58E-08 \| 1.22E-07 \| 5.82E-09 \| \| 6.23E-08 \| 1.27E-07 \| 3.49E-09 \| \| 4.55E-08 \| 8.28E-08 \| 1.48E-09 \| \| 3.86E-08 \| 6.42E-08 \| 1.86E-08 \| \| 6.37E-08 \| 5.19E-08 \| 5.86E-09 \| \| 3.53E-07 \| 2.51E-07 \| 8.24E-08 \| \| 2.94E-07 \| 3.11E-07 \| 2.73E-08 \| \| 2.86E-07 \| 1.72E-07 \| 8.67E-08 \| \| 4.76E-07 \| 4.06E-07 \| 2.80E-08 \| |

| EGFP-(s)-Spc24/mCherry-(s)-CENP-T | EGFP-(s)-CENP-S/mCherry-(s)-CENP-T |  |
| --- | --- | --- |
| Concentrations (R) (G) (RG) | Concentrations (R) (G) (RG) |  |
| \| 1.54E-07 \| 6.28E-08 \| 6.31E-09 \| \| --- \| --- \| --- \| \| 1.03E-07 \| 6.98E-08 \| 8.39E-09 \| \| 1.30E-07 \| 5.98E-08 \| 5.64E-08 \| \| 8.31E-07 \| 3.35E-07 \| 7.51E-08 \| \| 6.74E-07 \| 5.32E-07 \| 5.08E-08 \| \| 1.14E-06 \| 2.42E-07 \| 1.19E-07 \| \| 6.91E-07 \| 3.60E-07 \| 1.28E-07 \| \| 1.58E-06 \| 7.94E-07 \| 7.01E-07 \| \| 2.24E-07 \| 1.73E-07 \| 6.39E-10 \| \| 1.70E-07 \| 1.25E-07 \| 1.56E-08 \| \| 1.93E-07 \| 8.39E-08 \| 7.57E-09 \| \| 1.29E-07 \| 8.83E-08 \| 4.02E-08 \| \| 2.61E-07 \| 1.05E-07 \| 4.16E-09 \| \| 1.91E-07 \| 9.83E-08 \| 1.80E-08 \| \| 8.71E-08 \| 9.99E-08 \| 9.02E-09 \| \| 8.03E-08 \| 1.02E-07 \| 4.67E-09 \| \| 7.37E-08 \| 7.25E-08 \| 3.36E-09 \| \| 4.83E-08 \| 6.69E-08 \| 1.03E-09 \| \| 2.12E-07 \| 2.14E-07 \| 2.01E-08 \| \| 1.57E-07 \| 1.70E-07 \| 2.32E-08 \| \| 5.57E-07 \| 4.34E-07 \| 9.71E-09 \| \| 3.16E-07 \| 3.24E-07 \| 2.23E-08 \| \| 4.38E-07 \| 2.18E-07 \| 3.60E-08 \| \| 4.71E-07 \| 4.14E-07 \| 5.39E-09 \| \| 5.46E-07 \| 7.53E-07 \| 4.53E-08 \| \| 3.91E-07 \| 2.44E-07 \| 1.51E-08 \| \| 3.39E-07 \| 4.22E-07 \| 3.62E-08 \| \| 4.41E-07 \| 1.53E-07 \| 7.28E-09 \| \| 3.25E-07 \| 1.48E-07 \| 1.64E-08 \| | \| 3.15E-07 \| 1.09E-07 \| 1.39E-08 \| \| --- \| --- \| --- \| \| 2.20E-07 \| 1.05E-07 \| 2.80E-08 \| \| 3.15E-07 \| 6.72E-08 \| 3.28E-08 \| \| 3.48E-07 \| 1.29E-07 \| 6.62E-08 \| \| 2.96E-07 \| 2.63E-07 \| 1.85E-08 \| \| 3.66E-07 \| 2.72E-07 \| 5.55E-08 \| \| 2.73E-07 \| 1.26E-07 \| 2.12E-09 \| \| 3.73E-07 \| 3.49E-07 \| 2.17E-09 \| \| 4.84E-07 \| 5.34E-07 \| 3.69E-09 \| \| 2.05E-06 \| 1.27E-06 \| 1.88E-07 \| \| 1.84E-06 \| 5.61E-07 \| 1.57E-07 \| \| 1.62E-06 \| 7.93E-07 \| 8.64E-08 \| \| 1.10E-07 \| 1.49E-07 \| 4.35E-09 \| \| 1.26E-07 \| 9.44E-08 \| 5.35E-09 \| \| 1.58E-07 \| 3.11E-08 \| 8.06E-10 \| \| 4.89E-07 \| 4.51E-07 \| 1.63E-08 \| \| 2.38E-07 \| 7.92E-07 \| 9.35E-08 \| \| 2.62E-07 \| 1.85E-07 \| 2.43E-07 \| \| 5.92E-07 \| 2.53E-07 \| 8.54E-08 \| \| 4.12E-07 \| 1.88E-07 \| 9.25E-09 \| \| 4.74E-06 \| 2.12E-06 \| 2.18E-07 \| \| 6.54E-06 \| 3.00E-06 \| 1.28E-07 \| \| 1.31E-06 \| 1.82E-06 \| 8.18E-08 \| \| 1.03E-06 \| 1.88E-06 \| 4.24E-08 \| \| 1.16E-06 \| 6.96E-07 \| 1.07E-07 \| \| 9.91E-07 \| 1.23E-06 \| 1.54E-07 \| \| 1.54E-06 \| 9.23E-07 \| 4.11E-07 \| \| 1.01E-06 \| 6.46E-07 \| 2.33E-08 \| \| 4.49E-07 \| 1.19E-06 \| 6.44E-07 \| \| 7.54E-07 \| 1.17E-06 \| 8.66E-08 \| \| 6.56E-07 \| 6.30E-07 \| 9.77E-10 \| \| 5.94E-07 \| 5.36E-07 \| 1.19E-09 \| \| 5.84E-07 \| 5.09E-07 \| 2.49E-08 \| \| 1.17E-06 \| 3.63E-07 \| 1.48E-08 \| \| 6.16E-07 \| 4.60E-07 \| 3.73E-09 \| \| 2.47E-06 \| 2.09E-06 \| 1.50E-07 \| \| 2.94E-06 \| 4.23E-06 \| 3.84E-07 \| \| 1.26E-06 \| 2.01E-06 \| 2.23E-07 \| \| 2.28E-06 \| 8.75E-07 \| 6.76E-09 \| \| 1.33E-06 \| 9.94E-07 \| 2.39E-07 \| \| 1.53E-06 \| 6.52E-07 \| 7.89E-08 \| \| 1.88E-06 \| 8.34E-07 \| 1.31E-07 \| \| 1.68E-06 \| 6.12E-07 \| 1.05E-07 \| \| 2.39E-06 \| 3.39E-07 \| 5.19E-07 \| \| 2.59E-06 \| 7.70E-07 \| 7.76E-08 \| \| 2.66E-07 \| 3.70E-07 \| 1.51E-08 \| \| 5.27E-06 \| 2.96E-06 \| 3.59E-07 \| \| 3.10E-06 \| 1.27E-06 \| 2.41E-06 \| \| 4.85E-06 \| 2.50E-06 \| 1.36E-07 \| \| 2.69E-06 \| 2.40E-06 \| 1.88E-06 \| \| 2.99E-06 \| 2.39E-06 \| 9.06E-07 \| \| 2.80E-06 \| 2.01E-06 \| 3.18E-06 \| \| 1.86E-06 \| 6.46E-07 \| 1.17E-08 \| \| 6.14E-07 \| 3.99E-07 \| 1.86E-08 \| \| 4.73E-07 \| 3.58E-07 \| 1.91E-08 \| \| 9.58E-07 \| 2.45E-07 \| 9.05E-09 \| \| 8.95E-07 \| 1.35E-06 \| 6.34E-08 \| \| 8.26E-07 \| 1.21E-06 \| 1.57E-07 \| \| 1.30E-06 \| 1.02E-06 \| 1.50E-07 \| \| 4.05E-06 \| 2.68E-06 \| 6.72E-08 \| \| 2.67E-06 \| 5.71E-06 \| 1.15E-06 \| |  |

Corrected concentrations

| EGFP-(s)-CENP-S/mCherry-(s)-CENP-X | EGFP-(s)-CENP-W/mCherry-(s)-CENP-T | EGFP-(s)-CENP-T^ΔN^/mCherry-(s)-CENP-W |
| --- | --- | --- |
| Concentrations (R) (G) (RG) | Concentrations (R) (G) (RG) | Concentrations (R) (G) (RG) |
| \| 3.45E-07 \| 1.13E-07 \| 1.64E-07 \| \| --- \| --- \| --- \| \| 1.01E-07 \| 3.86E-07 \| 2.78E-07 \| \| 8.22E-08 \| 1.44E-07 \| 7.63E-08 \| \| 1.07E-07 \| 9.67E-08 \| 9.58E-08 \| \| 3.36E-08 \| 3.49E-08 \| 2.76E-08 \| \| 1.55E-07 \| 1.77E-07 \| 3.73E-08 \| \| 3.91E-07 \| 2.73E-07 \| 3.01E-07 \| \| 4.35E-07 \| 2.83E-07 \| 2.38E-07 \| \| 2.98E-07 \| 6.48E-07 \| 4.55E-07 \| \| 3.16E-07 \| 2.89E-07 \| 3.14E-07 \| \| 9.97E-08 \| 3.90E-07 \| 3.02E-07 \| \| 9.83E-08 \| 3.34E-07 \| 1.69E-07 \| \| 5.58E-08 \| 3.53E-07 \| 2.09E-07 \| \| 4.26E-09 \| 4.89E-07 \| 2.21E-07 \| \| 3.32E-07 \| 2.79E-07 \| 2.34E-07 \| \| 5.88E-07 \| 2.79E-07 \| 4.39E-07 \| \| 2.20E-07 \| 3.12E-07 \| 1.74E-07 \| \| 7.98E-08 \| 4.30E-07 \| 3.13E-07 \| \| 3.11E-07 \| 2.36E-07 \| 5.56E-07 \| \| 2.02E-07 \| 4.43E-07 \| 3.68E-07 \| \| 2.47E-07 \| 3.06E-07 \| 4.39E-07 \| \| 2.04E-07 \| 3.48E-07 \| 3.23E-07 \| \| 1.09E-07 \| 2.38E-07 \| 2.72E-07 \| \| 2.50E-07 \| 4.04E-07 \| 3.37E-07 \| \| 1.91E-07 \| 4.34E-07 \| 3.23E-07 \| \| 1.85E-07 \| 3.76E-07 \| 3.07E-07 \| \| 2.36E-07 \| 4.61E-07 \| 4.52E-07 \| \| 2.69E-07 \| 4.06E-07 \| 3.53E-07 \| \| 2.83E-07 \| 3.23E-07 \| 3.56E-07 \| \| 1.32E-07 \| 5.14E-07 \| 3.24E-07 \| \| 2.09E-07 \| 1.64E-07 \| 1.31E-07 \| \| 2.41E-07 \| 8.42E-08 \| 1.15E-07 \| \| 4.85E-07 \| 2.07E-07 \| 1.33E-07 \| \| 3.28E-07 \| 2.80E-07 \| 3.24E-07 \| \| 2.83E-07 \| 3.23E-07 \| 3.56E-07 \| \| 3.96E-07 \| 2.80E-07 \| 2.99E-07 \| \| 3.43E-07 \| 2.64E-07 \| 3.01E-07 \| \| 9.98E-08 \| 9.54E-08 \| 1.15E-07 \| \| 3.91E-07 \| 2.73E-07 \| 3.01E-07 \| \| 7.94E-08 \| 1.42E-07 \| 7.91E-08 \| \| 9.89E-08 \| 1.04E-07 \| 1.09E-07 \| \| 2.02E-07 \| 3.45E-07 \| 1.93E-07 \| \| 2.41E-07 \| 4.14E-07 \| 3.74E-07 \| \| 2.98E-08 \| 3.82E-08 \| 3.38E-08 \| \| 1.68E-09 \| 8.91E-09 \| 1.51E-08 \| \| 1.66E-07 \| 1.81E-07 \| 3.18E-08 \| \| 4.06E-07 \| 2.62E-07 \| 3.07E-07 \| \| 3.92E-07 \| 3.14E-07 \| 2.24E-07 \| \| 3.23E-07 \| 6.20E-07 \| 4.30E-07 \| \| 3.07E-07 \| 2.79E-07 \| 3.32E-07 \| \| 8.95E-08 \| 3.90E-07 \| 3.31E-07 \| | \| 4.31E-07 \| 4.89E-07 \| 1.02E-07 \| \| --- \| --- \| --- \| \| 6.99E-07 \| 1.86E-06 \| 2.92E-07 \| \| 4.92E-07 \| 3.34E-07 \| 1.97E-07 \| \| 4.61E-07 \| 2.61E-07 \| 1.40E-07 \| \| 4.04E-07 \| 3.74E-07 \| 1.17E-08 \| \| 2.81E-07 \| 3.29E-07 \| 1.43E-07 \| \| 4.03E-07 \| 2.75E-07 \| 1.72E-07 \| \| 4.40E-07 \| 2.22E-07 \| 2.56E-07 \| \| 3.54E-07 \| 2.56E-07 \| 2.18E-07 \| \| 3.48E-07 \| 2.87E-07 \| 4.79E-08 \| \| 3.91E-07 \| 1.69E-07 \| 2.26E-07 \| \| 1.68E-09 \| 1.60E-07 \| 2.42E-07 \| \| 1.07E-07 \| 2.22E-07 \| 3.13E-07 \| \| 1.67E-07 \| 2.42E-07 \| 6.26E-07 \| \| 3.15E-07 \| 2.70E-07 \| 3.99E-07 \| \| 2.08E-07 \| 3.05E-07 \| 8.74E-08 \| \| 1.69E-07 \| 3.63E-07 \| 1.61E-07 \| \| 1.40E-07 \| 2.96E-07 \| 1.66E-07 \| \| 1.43E-07 \| 2.73E-07 \| 1.24E-07 \| \| 1.80E-07 \| 2.47E-07 \| 9.54E-08 \| \| 7.06E-07 \| 5.31E-07 \| 7.97E-08 \| \| 4.80E-07 \| 3.69E-07 \| 2.70E-07 \| \| 4.28E-07 \| 3.33E-07 \| 2.31E-07 \| \| 4.20E-07 \| 9.16E-07 \| 4.20E-07 \| \| 5.79E-07 \| 4.23E-07 \| 4.47E-07 \| \| 1.42E-07 \| 3.06E-07 \| 8.59E-08 \| \| 1.30E-07 \| 3.29E-07 \| 1.35E-07 \| \| 1.02E-07 \| 2.41E-07 \| 1.75E-07 \| \| 3.93E-07 \| 2.40E-06 \| 3.76E-07 \| \| 2.24E-07 \| 2.97E-07 \| 1.66E-07 \| \| 7.21E-08 \| 2.17E-07 \| 2.58E-07 \| \| 8.52E-08 \| 1.70E-07 \| 2.09E-07 \| \| 6.65E-08 \| 1.49E-07 \| 1.85E-07 \| \| 1.39E-07 \| 1.43E-07 \| 1.60E-07 \| \| 5.53E-08 \| 3.76E-07 \| 2.60E-07 \| \| 3.39E-07 \| 1.85E-07 \| 2.26E-07 \| \| 2.07E-06 \| 1.57E-07 \| 1.77E-08 \| \| 1.60E-07 \| 1.43E-06 \| 3.45E-07 \| \| 3.07E-07 \| 4.78E-07 \| 2.56E-07 \| \| 1.16E-07 \| 1.52E-07 \| 4.24E-08 \| \| 9.83E-08 \| 1.24E-07 \| 5.84E-08 \| \| 2.55E-07 \| 1.28E-07 \| 1.14E-07 \| \| 2.34E-07 \| 3.12E-07 \| 1.65E-07 \| \| 8.78E-08 \| 3.58E-07 \| 1.21E-07 \| \| 8.88E-08 \| 3.16E-07 \| 9.13E-08 \| \| 9.72E-08 \| 2.67E-07 \| 9.02E-08 \| \| 8.74E-08 \| 1.59E-07 \| 5.25E-08 \| \| 9.02E-08 \| 9.69E-08 \| 3.75E-08 \| \| 8.76E-08 \| 2.03E-07 \| 5.49E-08 \| \| 9.54E-08 \| 1.68E-07 \| 6.80E-08 \| \| 4.85E-07 \| 6.21E-07 \| 2.29E-07 \| \| 5.80E-07 \| 8.01E-07 \| 2.60E-07 \| \| 2.77E-07 \| 2.11E-06 \| 5.35E-07 \| \| 4.66E-08 \| 1.53E-07 \| 7.66E-08 \| \| 5.44E-08 \| 9.44E-08 \| 2.39E-08 \| \| 4.20E-08 \| 6.79E-08 \| 3.00E-08 \| \| 4.60E-08 \| 5.33E-08 \| 3.28E-08 \| \| 2.97E-08 \| 5.95E-08 \| 3.13E-08 \| \| 3.24E-08 \| 5.96E-08 \| 3.05E-08 \| \| 1.15E-08 \| 2.05E-07 \| 1.91E-07 \| \| 7.41E-08 \| 1.39E-07 \| 1.15E-07 \| \| 4.97E-08 \| 1.10E-07 \| 1.25E-07 \| \| 5.45E-08 \| 1.04E-07 \| 1.07E-07 \| \| 1.07E-08 \| 1.33E-07 \| 1.44E-07 \| \| 3.36E-07 \| 4.48E-07 \| 2.77E-07 \| \| 2.10E-07 \| 8.33E-07 \| 2.39E-07 \| \| 1.35E-07 \| 6.75E-07 \| 2.65E-07 \| \| 1.92E-07 \| 4.82E-07 \| 2.31E-07 \| \| 2.05E-07 \| 4.82E-07 \| 2.28E-07 \| \| 2.18E-07 \| 5.51E-07 \| 2.58E-07 \| \| 4.14E-08 \| 8.26E-07 \| 4.52E-07 \| \| 1.91E-07 \| 1.34E-06 \| 5.68E-07 \| \| 8.44E-08 \| 5.36E-07 \| 1.78E-07 \| \| 3.01E-08 \| 8.07E-08 \| 1.14E-08 \| \| 3.85E-08 \| 4.29E-08 \| 2.15E-08 \| \| 2.40E-08 \| 5.71E-08 \| 7.86E-09 \| \| 2.24E-08 \| 5.49E-08 \| 1.71E-08 \| \| 9.98E-09 \| 5.00E-08 \| 2.05E-08 \| \| 1.10E-08 \| 3.91E-08 \| 2.38E-08 \| \| 6.58E-09 \| 9.86E-08 \| 3.58E-08 \| \| 7.75E-09 \| 5.43E-08 \| 1.61E-08 \| \| 1.32E-07 \| 2.40E-07 \| 2.24E-07 \| \| 1.64E-07 \| 2.22E-07 \| 1.85E-07 \| \| 1.78E-07 \| 1.59E-07 \| 1.25E-07 \| \| 1.86E-07 \| 9.94E-08 \| 1.55E-07 \| \| 2.68E-07 \| 8.34E-08 \| 1.23E-07 \| \| 3.08E-07 \| 1.20E-07 \| 1.31E-07 \| \| 2.18E-07 \| 1.75E-07 \| 1.23E-07 \| \| 4.84E-07 \| 2.62E-07 \| 2.60E-07 \| \| 3.97E-07 \| 4.12E-07 \| 2.34E-07 \| \| 3.64E-07 \| 1.99E-07 \| 2.43E-07 \| \| 2.27E-07 \| 1.93E-07 \| 1.49E-07 \| \| 2.23E-07 \| 2.07E-07 \| 1.57E-07 \| \| 8.37E-08 \| 4.44E-07 \| 3.83E-07 \| \| 2.10E-07 \| 3.69E-07 \| 2.74E-07 \| \| 3.15E-07 \| 1.68E-07 \| 2.78E-07 \| \| 2.46E-07 \| 1.22E-07 \| 2.27E-07 \| \| 7.41E-07 \| 3.83E-07 \| 3.07E-07 \| \| 9.60E-08 \| 1.14E-06 \| 2.74E-07 \| \| 1.55E-07 \| 9.32E-07 \| 2.92E-07 \| \| 4.21E-07 \| 1.17E-06 \| 3.33E-07 \| \| 3.87E-07 \| 1.20E-06 \| 3.24E-07 \| \| 3.51E-07 \| 8.44E-07 \| 2.73E-07 \| \| 3.38E-07 \| 7.36E-07 \| 2.49E-07 \| \| 3.33E-07 \| 6.66E-07 \| 2.72E-07 \| \| 1.41E-07 \| 3.24E-07 \| 1.30E-07 \| \| 1.68E-07 \| 2.24E-07 \| 7.75E-08 \| \| 2.87E-07 \| 1.57E-07 \| 1.14E-07 \| \| 3.82E-08 \| 2.14E-07 \| 1.54E-07 \| \| 8.12E-08 \| 1.59E-07 \| 1.05E-07 \| \| 7.80E-07 \| 7.28E-07 \| 2.23E-07 \| \| 2.33E-07 \| 6.47E-07 \| 2.95E-07 \| \| 8.89E-08 \| 6.82E-07 \| 3.23E-07 \| \| 2.99E-07 \| 6.23E-07 \| 2.99E-07 \| \| 1.58E-07 \| 5.48E-07 \| 3.72E-07 \| \| 1.44E-07 \| 3.95E-07 \| 2.57E-07 \| \| 2.29E-07 \| 3.38E-07 \| 2.08E-07 \| \| 2.14E-07 \| 3.56E-07 \| 1.53E-07 \| \| 2.60E-07 \| 3.11E-07 \| 2.97E-07 \| \| 1.46E-07 \| 2.12E-07 \| 2.81E-07 \| \| 4.32E-07 \| 4.25E-07 \| 5.08E-07 \| \| 6.12E-07 \| 3.88E-07 \| 3.16E-07 \| \| 4.69E-07 \| 3.23E-07 \| 2.39E-07 \| \| 4.12E-07 \| 2.63E-07 \| 1.85E-07 \| \| 3.42E-07 \| 2.18E-07 \| 1.89E-07 \| | \| 5.94E-07 \| 1.37E-06 \| 4.63E-07 \| \| --- \| --- \| --- \| \| 1.67E-07 \| 1.06E-07 \| 1.51E-08 \| \| 1.39E-07 \| 1.90E-07 \| 3.26E-08 \| \| 1.43E-07 \| 1.67E-07 \| 3.32E-08 \| \| 1.01E-07 \| 1.73E-07 \| 2.26E-08 \| \| 1.17E-07 \| 1.43E-07 \| 1.40E-08 \| \| 1.57E-07 \| 5.49E-08 \| 4.95E-10 \| \| 9.82E-08 \| 9.11E-08 \| 3.61E-09 \| \| 9.97E-08 \| 6.72E-08 \| 8.93E-09 \| \| 1.32E-07 \| 1.42E-07 \| 6.58E-09 \| \| 1.66E-07 \| 9.49E-08 \| 1.49E-08 \| \| 1.06E-07 \| 1.21E-07 \| 3.67E-08 \| \| 1.30E-07 \| 1.18E-07 \| 2.84E-08 \| \| 2.13E-07 \| 9.68E-08 \| 6.06E-08 \| \| 8.47E-08 \| 2.10E-07 \| 5.67E-09 \| \| 1.49E-07 \| 6.82E-08 \| 4.01E-08 \| \| 1.24E-07 \| 1.50E-07 \| 7.04E-09 \| \| 8.52E-08 \| 1.01E-07 \| 2.22E-08 \| \| 8.90E-08 \| 1.16E-07 \| 9.96E-09 \| \| 7.27E-08 \| 1.32E-07 \| 2.37E-08 \| \| 3.22E-07 \| 5.05E-07 \| 2.45E-07 \| \| 4.11E-07 \| 4.41E-07 \| 1.19E-07 \| \| 3.27E-07 \| 4.80E-07 \| 1.38E-07 \| \| 3.35E-07 \| 3.90E-07 \| 8.75E-08 \| \| 2.22E-07 \| 1.12E-07 \| 7.25E-09 \| \| 7.33E-08 \| 1.39E-07 \| 2.27E-09 \| \| 6.90E-08 \| 1.17E-07 \| 3.01E-09 \| \| 2.33E-08 \| 1.16E-07 \| 4.49E-08 \| \| 6.02E-08 \| 9.81E-08 \| 2.12E-08 \| \| 2.86E-07 \| 4.64E-07 \| 2.17E-07 \| \| 2.45E-07 \| 1.24E-07 \| 3.90E-07 \| \| 2.14E-07 \| 3.16E-07 \| 2.15E-07 \| \| 1.56E-06 \| 4.76E-07 \| 1.91E-07 \| \| 1.83E-06 \| 1.22E-08 \| 1.45E-07 \| \| 5.39E-07 \| 5.27E-07 \| 2.40E-07 \| \| 4.66E-07 \| 7.66E-07 \| 1.25E-07 \| \| 6.88E-07 \| 4.96E-07 \| 4.31E-07 \| \| 6.16E-07 \| 2.52E-07 \| 1.06E-07 \| \| 6.11E-07 \| 4.15E-07 \| 5.37E-08 \| \| 4.05E-07 \| 2.81E-07 \| 3.79E-08 \| \| 4.00E-07 \| 4.37E-07 \| 3.96E-08 \| \| 3.01E-07 \| 2.78E-07 \| 1.50E-07 \| \| 2.55E-07 \| 3.30E-07 \| 9.77E-08 \| \| 6.95E-07 \| 7.56E-07 \| 3.36E-07 \| \| 5.58E-08 \| 9.91E-08 \| 1.60E-08 \| \| 5.71E-08 \| 1.03E-07 \| 2.69E-08 \| \| 5.40E-08 \| 9.56E-08 \| 3.49E-08 \| \| 3.41E-08 \| 6.11E-08 \| 6.42E-08 \| \| 7.10E-08 \| 8.29E-08 \| 5.23E-08 \| \| 5.38E-08 \| 7.33E-08 \| 6.96E-08 \| \| 4.44E-08 \| 6.50E-08 \| 2.88E-08 \| \| 1.93E-07 \| 9.60E-08 \| 7.58E-09 \| \| 1.79E-07 \| 7.46E-08 \| 4.69E-08 \| \| 1.64E-07 \| 9.79E-08 \| 3.40E-09 \| \| 1.14E-07 \| 5.31E-08 \| 4.42E-08 \| \| 4.99E-08 \| 5.62E-08 \| 1.36E-08 \| \| 8.55E-08 \| 5.25E-08 \| 1.33E-08 \| \| 4.49E-08 \| 1.29E-07 \| 6.89E-08 \| \| 7.15E-08 \| 1.04E-07 \| 2.43E-08 \| \| 4.96E-08 \| 2.74E-08 \| 8.52E-09 \| \| 1.70E-07 \| 1.69E-07 \| 1.65E-08 \| \| 1.57E-07 \| 6.70E-08 \| 3.58E-08 \| \| 1.75E-07 \| 8.98E-08 \| 6.95E-09 \| \| 1.32E-07 \| 7.52E-08 \| 1.59E-08 \| \| 1.46E-07 \| 6.87E-08 \| 1.57E-08 \| \| 1.23E-07 \| 6.34E-08 \| 8.59E-09 \| \| 4.96E-08 \| 3.50E-08 \| 5.82E-09 \| \| 4.76E-08 \| 1.79E-08 \| 2.82E-09 \| \| 2.21E-08 \| 4.18E-08 \| 9.31E-09 \| \| 2.84E-08 \| 3.33E-08 \| 3.28E-09 \| \| 2.41E-08 \| 3.20E-08 \| 6.68E-09 \| \| 2.99E-08 \| 3.95E-08 \| 2.20E-08 \| \| 6.08E-07 \| 3.05E-07 \| 3.82E-08 \| \| 9.55E-07 \| 8.23E-07 \| 1.18E-07 \| \| 7.16E-07 \| 2.98E-07 \| 1.00E-08 \| \| 1.18E-06 \| 4.85E-07 \| 9.45E-08 \| \| 1.59E-07 \| 3.41E-07 \| 3.50E-09 \| \| 1.24E-07 \| 3.77E-07 \| 3.44E-08 \| \| 1.21E-07 \| 3.06E-07 \| 3.81E-08 \| \| 1.30E-07 \| 1.22E-07 \| 8.12E-09 \| \| 1.41E-07 \| 1.00E-07 \| 6.66E-09 \| \| 7.05E-07 \| 6.45E-07 \| 8.04E-08 \| \| 5.66E-07 \| 3.18E-07 \| 2.72E-07 \| \| 5.74E-07 \| 4.83E-07 \| 1.00E-07 \| \| 4.04E-07 \| 6.06E-07 \| 1.68E-07 \| \| 4.13E-07 \| 2.04E-07 \| 3.57E-08 \| \| 3.49E-07 \| 2.09E-07 \| 3.62E-08 \| \| 1.83E-07 \| 1.16E-07 \| 1.50E-07 \| \| 3.77E-07 \| 1.02E-07 \| 6.61E-08 \| \| 2.39E-07 \| 1.96E-07 \| 7.90E-08 \| \| 1.68E-07 \| 3.09E-07 \| 5.88E-08 \| \| 3.05E-07 \| 2.47E-07 \| 2.11E-08 \| \| 1.60E-07 \| 2.35E-07 \| 6.09E-08 \| \| 2.39E-07 \| 1.61E-07 \| 3.97E-08 \| \| 2.27E-07 \| 1.12E-07 \| 1.65E-08 \| \| 1.90E-07 \| 3.58E-07 \| 4.41E-08 \| \| 2.28E-07 \| 1.29E-07 \| 4.89E-08 \| \| 2.41E-07 \| 1.68E-07 \| 6.42E-08 \| \| 1.49E-07 \| 1.63E-07 \| 4.51E-08 \| \| 3.00E-07 \| 1.68E-07 \| 6.53E-08 \| \| 2.91E-07 \| 2.21E-07 \| 1.70E-08 \| \| 2.09E-07 \| 1.89E-07 \| 3.34E-08 \| \| 1.84E-07 \| 6.74E-08 \| 8.96E-08 \| \| 1.58E-07 \| 1.52E-07 \| 1.45E-07 \| \| 1.05E-07 \| 2.15E-07 \| 4.79E-08 \| \| 1.35E-07 \| 1.43E-07 \| 3.72E-08 \| \| 2.86E-07 \| 2.48E-07 \| 1.59E-08 \| \| 1.25E-07 \| 8.71E-08 \| 9.63E-08 \| \| 5.93E-08 \| 9.28E-08 \| 5.36E-08 \| \| 7.86E-07 \| 1.08E-06 \| 3.17E-07 \| \| 1.06E-06 \| 6.55E-07 \| 3.63E-08 \| \| 1.12E-06 \| 6.62E-07 \| 9.65E-08 \| \| 1.07E-06 \| 3.72E-07 \| 5.04E-07 \| \| 1.04E-06 \| 1.37E-06 \| 2.77E-07 \| \| 5.66E-07 \| 7.11E-07 \| 2.48E-08 \| \| 6.18E-07 \| 4.10E-07 \| 3.50E-08 \| \| 5.24E-07 \| 4.83E-07 \| 1.42E-07 \| \| 7.51E-07 \| 3.18E-07 \| 6.38E-08 \| \| 6.63E-07 \| 3.95E-07 \| 4.13E-09 \| \| 2.07E-07 \| 1.43E-07 \| 2.67E-08 \| \| 4.08E-07 \| 2.54E-07 \| 7.21E-09 \| \| 4.01E-07 \| 5.31E-07 \| 7.43E-09 \| \| 3.47E-07 \| 5.01E-07 \| 9.91E-08 \| \| 2.30E-07 \| 4.32E-07 \| 7.96E-08 \| \| 3.01E-07 \| 2.10E-07 \| 8.26E-08 \| \| 2.74E-07 \| 2.95E-07 \| 6.07E-08 \| \| 3.45E-07 \| 1.32E-07 \| 1.89E-08 \| \| 2.25E-07 \| 2.32E-07 \| 8.09E-08 \| \| 1.56E-07 \| 2.11E-07 \| 1.88E-08 \| \| 2.64E-07 \| 1.85E-07 \| 1.67E-08 \| \| 1.73E-07 \| 2.66E-07 \| 9.66E-09 \| \| 1.50E-07 \| 2.04E-07 \| 4.16E-08 \| \| 1.00E-07 \| 2.19E-07 \| 4.82E-08 \| \| 1.75E-07 \| 1.53E-07 \| 8.30E-09 \| \| 9.46E-08 \| 1.34E-07 \| 2.45E-08 \| \| 6.81E-08 \| 2.06E-07 \| 9.76E-08 \| \| 1.28E-07 \| 3.21E-07 \| 1.33E-07 \| |
